# Quantitative Characterization of Three Carbonic Anhydrase Inhibitors by LESA Mass Spectrometry

**DOI:** 10.1021/jasms.2c00024

**Published:** 2022-06-08

**Authors:** Eva Illes-Toth, Christopher J. Stubbs, Emma K. Sisley, Jeddidiah Bellamy-Carter, Anna L. Simmonds, Todd H. Mize, Iain B. Styles, Richard J. A. Goodwin, Helen J. Cooper

**Affiliations:** † School of Biosciences, University of Birmingham, Birmingham B15 2TT, United Kingdom; ‡ Mechanistic and Structural Biology, Discovery Sciences, R&D, AstraZeneca, Cambridge CB4 0WG, United Kingdom; § School of Computer Science and Centre of Membrane Proteins and Receptors (COMPARE), University of Birmingham, Birmingham B15 2TT, United Kingdom; ∥ The Alan Turing Institute, London NW1 2DB, United Kingdom; ⊥ University of Nottingham, Midlands NG7 2RD, United Kingdom; # Imaging and Data Analytics, Clinical Pharmacology & Safety Sciences, BioPharmaceuticals R&D, AstraZeneca, Cambridge CB4 0WG, United Kingdom

## Abstract

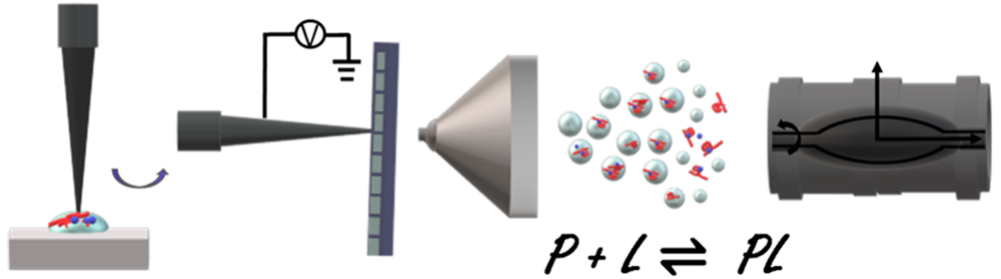

Liquid extraction surface analysis (LESA) coupled to native mass spectrometry (MS) presents unique analytical opportunities due to its sensitivity, speed, and automation. Here, we examine whether this tool can be used to quantitatively probe protein–ligand interactions through calculation of equilibrium dissociation constants (*K*_d_ values). We performed native LESA MS analyses for a well-characterized system comprising bovine carbonic anhydrase II and the ligands chlorothiazide, dansylamide, and sulfanilamide, and compared the results with those obtained from direct infusion mass spectrometry and surface plasmon resonance measurements. Two LESA approaches were considered: In one approach, the protein and ligand were premixed in solution before being deposited and dried onto a solid substrate for LESA sampling, and in the second, the protein alone was dried onto the substrate and the ligand was included in the LESA sampling solvent. Good agreement was found between the *K*_d_ values derived from direct infusion MS and LESA MS when the protein and ligand were premixed; however, *K*_d_ values determined from LESA MS measurements where the ligand was in the sampling solvent were inconsistent. Our results suggest that LESA MS is a suitable tool for quantitative analysis of protein–ligand interactions when the dried sample comprises both protein and ligand.

## Introduction

Protein–ligand binding is of key importance in all living organisms for the maintenance of protein structure and function, enzymatic activity, molecular interactions, signaling, and recognition. Therapeutic interventions exploit noncovalent interactions between the drug ligand and the target protein. Understanding the binding characteristics, e.g., stoichiometry, equilibrium dissociation constants, binding sites, and nature of binding (covalent vs noncovalent interactions), is therefore of paramount importance in the discovery and development of drugs. Detailed characterization of these essential parameters can be performed by an array of analytical techniques including mass spectrometry (MS),^1^ isothermal titration calorimetry (ITC),^2^ surface plasmon resonance (SPR),^3^ fluorescence spectroscopy, fluorescence correlation spectroscopy (FCS),^4^ and nuclear magnetic resonance (NMR).^5,6^

Native MS in conjunction with nanoelectrospray ionization (nESI), herein referred to as direct infusion for simplicity, has been widely exploited for the quantitative analysis of protein–ligand and protein–protein interactions,^7^ including for the characterization of protein–small molecule,^8−10^ protein–phosphopeptide,^11^ protein–carbohydrate,^12,13^ protein–protein,^14^ protein–DNA,^15^ and protein–RNA^16^ complexes. Broadly, the capabilities of direct infusion MS for the study of protein–ligand binding are twofold. First, the resultant mass shift, as detected in the mass spectrum, is indicative of binding of a ligand to a protein. Second, the relative intensities of unbound protein ion peaks and ligand-bound protein ion peaks can be used to quantitatively monitor the extent of binding. Typically, the concentration of the protein of interest is fixed, and the ligand concentration is varied in order to perform a titration. With increasing ligand concentrations, a proportional increase in the intensity of the ligand-bound peak is observed.^17^ The ratio of the protein–ligand complex and the protein, referred to as the response (*R*), is plotted as a function of initial ligand concentration.^15,18−20^ This plot serves as the basis for determination of the equilibrium dissociation constant (*K*_d_) via curve fitting with a linear^19^ or nonlinear function.^12^ The relative intensities or peak areas of the ligand-free and ligand-bound peaks are expressed for all observed charge states rather than for individual charge states in order to obtain a more comprehensive picture: individual charge states may disproportionally represent single, distinct conformational forms.^9,21^

Protein–ligand titrations monitored in the gas phase assume that the ionization efficiency of unbound protein and the protein–ligand complex is similar in the case of a small ligand that does not substantially increase the mass and that the initial protein concentration does not change during the ionization and detection process.^17,22^ Nevertheless, source conditions and instrumental parameters can affect the stability of complexes, necessitating cautious interpretation of the derived equilibrium dissociation constants.^11,12,14,19,23^ Minimization of in-source dissociation of low-affinity, labile complexes is a critical step of gas-phase titrations and can be experimentally achieved through optimal selection of tuning conditions, reducing the diameter of the electrospray emitter, and/or application of a postacquisition correction to account for partial in-source dissociation.^24,25^ The use of complementary in-solution techniques has proven to be helpful for further validation.^26,27^

Here, we sought to evaluate the use of liquid extraction surface analysis (LESA)^28^ MS for the measurement of binding affinities under native-like conditions. Native LESA MS appears to be an attractive strategy for the study of protein–ligand or protein–protein interactions as it affords automation prior to MS, allows direct sampling from a solid surface, and has the potential to lend itself to measurement of binding affinities in *ex vivo* tissue samples. Indeed, we have demonstrated analyses of endogenous protein assemblies and protein ligands from thin tissue sections by native LESA MS.^29−31^ As an initial step in exploring the utility of LESA for quantitative analysis of protein–ligand binding, we examine the utility of native LESA MS for the measurement of *K*_d_ values by use of a well-characterized model system comprising bovine carbonic anhydrase (CAH) and three ligands, chlorothiazide (CTZ), dansylamide (DNSA), and sulfanilamide (SLFA) (Figure S1, Supporting Information). In numerous previous biophysical studies, CTZ,^9,32^ DNSA,^32,33^ and SLFA^27,34,35^ have shown to be potent inhibitors of various isoforms and derivatives of CAH. The carbonic anhydrases are a large group of isoenzymes,^34,36^ some of which are membrane bound while others are cytosolic or secreted, that catalyze the interconversion of CO_2_ and bicarbonate.^37^ They represent key pharmaceutical targets owing to their involvement in infection, cancer, glaucoma, obesity, and hypertension.^36,38^ Bovine CAH is a particularly well-described model protein, a 259-residue enzyme^39^ that catalyzes the hydration of carbon dioxide and dehydration of bicarbonate.^37^ In its hydrophobic, active center it contains a Zn^2+^ ion coordinated by three imidazole moieties and a hydroxide group to which many of its inhibitors bind.^40−42^ Sulfonamides and their derivatives, e.g., SLFA and CTZ, bind to CAH via coordination to the Zn^2+^ in the center of the binding pocket.^36,40,43^ The aromatic rings of Zn^2+^ binders can occupy both the hydrophobic and the hydrophilic parts of the binding cavity, whereas the tails tend to orient toward the exit of the binding pocket.^36^ DNSA favors the hydrophobic region of the binding pocket with its naphthalene ring rotated in a 54° angle.^44^

We compared *K*_d_ values measured by direct infusion MS with those measured by LESA MS. Two LESA strategies were trialled. In the first, the protein and ligand were mixed in solution prior to deposition and drying on a foil-covered glass slide. The resulting dried protein/ligand spots were sampled by LESA using a solvent comprising 25 mM ammonium acetate. We refer to this approach as “LESA_premix_”. In the second, solutions of the pure protein were deposited and dried onto foil-covered glass slides. The dried protein spot was sampled with a solvent comprising 25 mM ammonium acetate which contained the ligand. We refer to this approach as “LESA_ligand_”. To corroborate our data, *K*_d_ values determined by MS were validated against SPR measurements. SPR is a label-free, surface-based, optical technique commonly deployed for the characterization of macromolecular interactions.^45,46^

## Experimental Section

### Sample Preparation

Ten micromolar bovine carbonic anhydrase II (CAH) was mixed with various concentrations of CTZ, DNSA, and SLFA in 25 mM ammonium acetate (pH 7.0), incubated for 10 min at room temperature (21–22 °C), and processed by direct infusion nESI MS or by native LESA MS. LESA was performed by use of the Triversa Nanomate platform (Advion, Ithaca, NY). Full details of the sampling procedures are described in the Supporting Information and summarized below.

#### LESA_premix_

A 1.5 μL amount of 10 μM CAH only or [CAH + ligand] was spotted onto a microscope glass slide covered with a layer of Al foil and allowed to air dry at room temperature. The dried spots were sampled with 25 mM ammonium acetate (pH 7.0) with a dwell time of 60 s following aspiration of 5.0 μL of solvent and deposition of 3.0 μL. A 3.5 μL amount was reaspirated to ensure complete sample collection.

#### LESA_ligand_

A 1.5 μL amount of 10 μM CAH was spotted onto a microscope glass slide covered with Al foil and allowed to air dry at room temperature. The dried spots were sampled with 25 mM ammonium acetate (pH 7.0) containing various concentrations of CTZ, DNSA, or SLFA at room temperature. The sampling protocol was comprised of 10 mix and repeat cycles at the settings above to control for the 10 min incubation used for LESA_premix_.

### Mass Spectrometry and Data Analyses

All protein–ligand titration experiments were performed on an Orbitrap Elite mass spectrometer (Thermo Fisher Scientific, Bremen, Germany) with an automatic gain control target of 1 × 10^6^ charges in full-scan mode, 250 °C source temperature, 25–30% RF voltage, 5 or 10 ms injection time, 1000 microscans, and 120 000 resolution. Data were acquired in triplicate in the 200–4000 *m*/*z* mass range for 3–5 min. The ion intensities of each acquisition were summed in Xcalibur (Foundation 3.0 SP2), written to a raw file, and further processed in MATLAB (version R2017a and statistics toolbox, Mathworks, Inc., Natick, MA) and Python (version 3.7.6) using in-house software (see Supplemental File 1, Supporting Information). Using MATLAB, the peak areas of protonated unbound and ligand-bound protein peaks in all observed charge states (9+, 10+, and 11+) were determined using the trapezium rule. The average ratio, *R*, of bound to unbound protein across the three charge states was determined. No baseline correction was performed, and only the main protonated ion was considered. Titration curves were then generated in Python. The mean fraction bound (*R*/(*R* + 1)) from experimental replicates was plotted as a function of the initial ligand concentration *L*_0_ (μM).

Equilibrium dissociation constants were then calculated by fitting the titration curves using a nonlinear sum of least squares. In the case of direct infusion measurements, equilibrium dissociation constants were calculated by fitting the titration curves to eq 1 (described in detail in ref (25)), where *P*_0_ denotes the initial protein concentration, *K*_d_ is the equilibrium dissociation constant, and *f*_sat_ is a correction factor to account for gas-phase dissociation and is the experimental bound fraction at saturation.
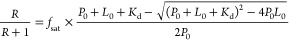
1For the LESA_premix_ and LESA_ligand_ measurements, corrected values of *L*_0_ and/or *P*_0_ were used to account for extraction efficiency and dilution effects associated with the sampling process as described below.

### Determination of *P*_0_ Correction for Combined Protein Extraction Efficiency and Dilution Effects

#### LESA_premix_

A paper grid print of a 1584-well plate was placed into the plate holder of the stage directly under the glass slide covered with Al foil containing the samples in order to guide manual selection of *xy* coordinates. In addition, a halved 96-well plate was placed beside the glass slide for collection of extracted aliquots. The advanced user interface (AUI) was used to aspirate 5 μL of 25 mM ammonium acetate and dispense that onto a 1.5 μL dried droplet of 10 μM CAH at a 3 μL/min flow rate, 1.4–1.6 mm height, mimicking the formation of a liquid junction at a 60 s dwell time. A 3.5 μL volume was reaspirated for collection into a half 96-well plate. For each location sampled (*n* = 15), two 1.2 μL samples were analyzed on the DeNovix DS-II spectrophotometer. The UV spectra at 280 nm (*A*_280_) were used to calculate the corrected concentration (in micromolar) based on the extinction coefficient of CAH (50 070 M^–1^ cm^–1^ ^47^). The absorption of 25 mM ammonium acetate was subtracted from that of CAH. Data were exported as .csv files, and the correction factor incorporating both the dilution effect and the extraction efficiency of LESA was calculated in Prism GraphPad 6.01. The mean concentration of 15 sample spots (micromolar) was used as the corrected value for *P*_0_.

#### LESA_ligand_

The corrected value for *P*_0_ was determined as above except that the sampling procedure was adjusted to mimic the LESA_ligand_ sampling procedure in which 10 mix and repeat steps were included. To aid sample collection, the blowout tab was checked in the software and the volume was released at a pressure of 3 psi and duration of 10 s. In total, 10 spots were sampled and analyzed as above. The mean concentration (micromolar) was used directly as the corrected value for *P*_0_.

### LESA Correction Factor for the Total Free Ligand Extraction Efficiency and Dilution Effects

The absorption spectra of different concentrations of CTZ, SLFA, and DNSA were obtained by spectrophotometry (wavelength 220–500 nm) for a solution reference curve without any sample manipulation, *n* = 3. The wavelength of maximum absorption was determined from these samples. A 1.5 μL amount of ligand was deposited on the surface of a glass slide covered with Al foil, air dried, placed on top of a 1584-well paper grid at different concentrations in order to determine its coordinates, and extracted in 25 mM ammonium acetate in triplicate using the aforementioned LESA AUI sampling conditions. The absorption spectra of the samples following LESA AUI were collected and exported as .csv files, after confirming no significant red or blue shift, in order to calculate the correction factor accounting for both dilution effects and extraction efficiency and any evaporation occurring during extended LESA sampling in the case of the LESA_ligand_ for the free ligands at different concentrations. The mean and standard deviation values were calculated in Prism GraphPad 6.01 based on the solution reference values and the AUI collected samples. The mean value ± STD of the entire concentration range measured yielded the correction factor used in the *K*_d_ calculations. The mean correction factor was used to multiply the initial reference *L*_0_ values to obtain its paired corrected *L*_0_.

### Traveling Wave Ion Mobility Spectrometry MS, Surface Plasmon Resonance, and Circular Dichroism Spectroscopy

Details are given in the Supporting Information.

## Results and Discussion

**Figure 1:**
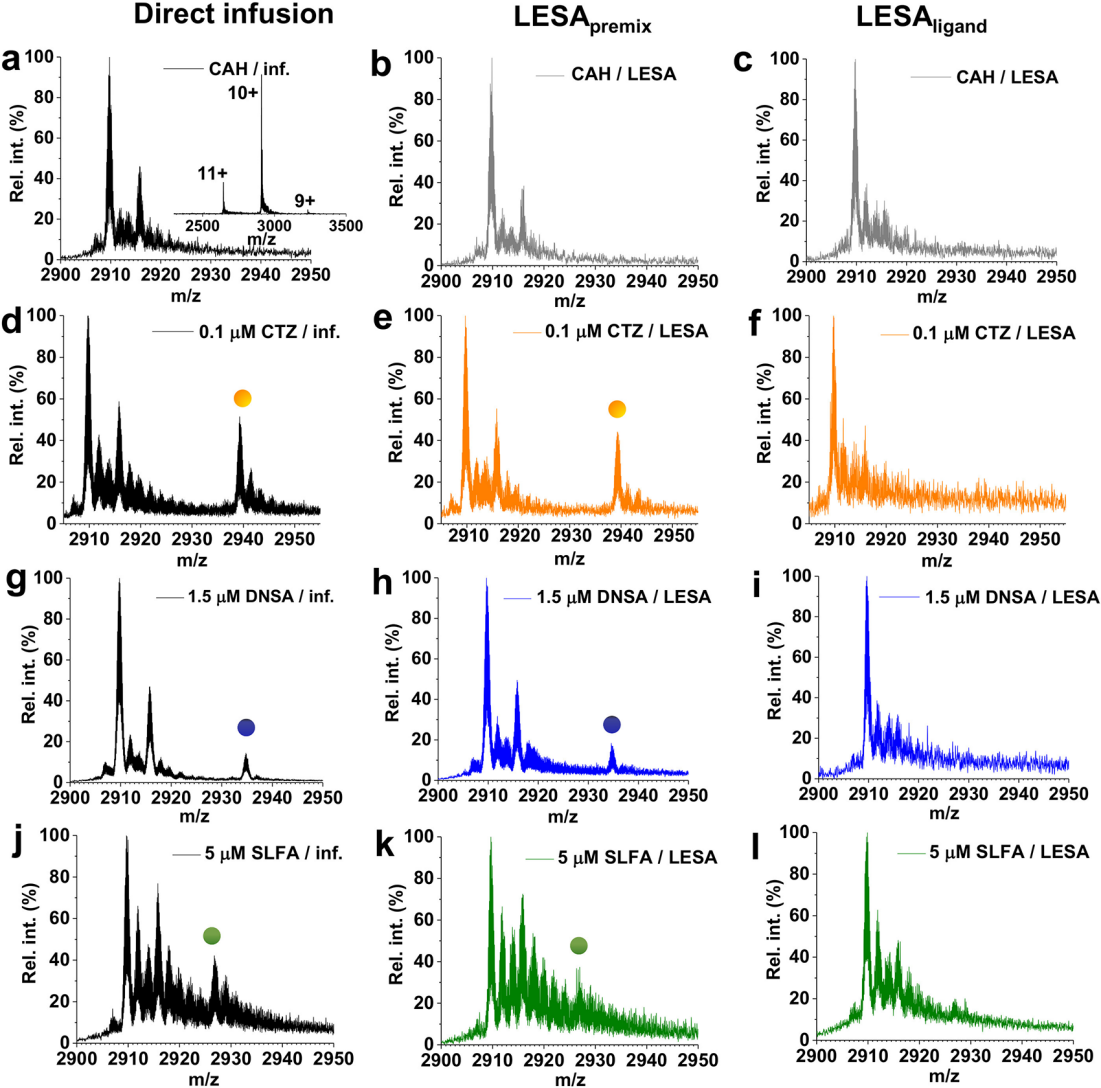
Native mass spectra of CAH following direct infusion (a), LESA_premix_ (b), and LESA_ligand_ (c) sampling. (Inset) CSD of CAH in its Zn^2+^-bound form with the 10^+^ ion being the most abundant. Native mass spectra of CAH in the presence of 0.1 μM CTZ following direct infusion (d), LESA_premix_ (e), and LESA_ligand_ (f). Mass spectra of CAH in the presence of 1.5 μM DNSA obtained in direct infusion mode (g) and subsequently LESA_premix_ (h) and LESA_ligand_ (i) sampling. Native mass spectra of CAH in the presence of 5 μM SLFA following direct infusion (j), LESA_premix_ (k), and LESA_ligand_ (l) sampling. Dots represent the protein–ligand complexes.

Figure 1 shows the mass spectra obtained following direct infusion, LESA_premix_, and LESA_ligand_ for CAH and the three ligands at various ligand concentrations (0, 0.1, 1.5, and 5 μM). For CAH, a typical narrow charge state distribution (CSD) was observed ranging from 9^+^ to 11^+^ with the 10^+^ charge state being the most dominant (Figure 1a–c), indicative of retention of its native structure. The deconvoluted mass, 29 088.86 Da, revealed that CAH was N-terminally acetylated with a Zn^2+^ ion bound to the protein (Figure S2, Supporting Information) and is in good agreement (<5 ppm) with the theoretically calculated mass of the protein with 29 088.78 Da (accession ID P00921).

In the presence of CTZ, a 295 Da mass shift was observed in the direct infusion (Figure 1d) and LESA_premix_ (Figure 1e) samples at concentrations of 0.1 μM and above. No obvious ligand binding was observed at 0.1 μM CTZ with LESA_ligand_ MS (Figure 1f) but was observed at concentrations above 1 μM (Figure S3, Supporting Information). For DNSA, ligand binding was observed in the mass spectra (Δ 250 Da) at 1.5 μM and above in both direct infusion (Figure 1g) and LESA_premix_ (Figure 1h). No ligand binding was observed following LESA_ligand_ (Figure 1i) at this concentration; however, it could be observed at concentrations above 3 μM (Figure S4, Supporting Information). In the presence of SLFA, the mass spectra revealed that a 172 Da mass shift, corresponding to ligand binding, occurred from a 5 μM concentration onward in direct infusion mode (Figure 1j) and in LESA_premix_ mode (Figure 1k), but a well-resolved peak for the protein–ligand complex could not be detected in LESA_ligand_ (Figure 1l) mode. For LESA_ligand_ mode, ligand binding was observed at concentrations of 10 μM and above (Figure S5, Supporting Information). There was no evidence for binding of multiple ligands at any concentration. No binding was observed for any of the ligands in the presence of 1% (v/v) formic acid (Figures S3–S5, Supporting Information).

The discrepancies in the concentrations at which the protein–ligand complexes were observed in the various sampling modes (direct infusion, LESA_premix_, and LESA_ligand_) together with the deviations in the observed relative abundance suggest that the extraction efficiency of LESA and the dilution inherent in the LESA sampling process result in changes to *P*_0_ and/or *L*_0_, thus shifting the equilibria described in eq 1. In order to calculate the *K*_d_ values from LESA data, it is necessary to determine the *P*_0_ and *L*_0_ values corrected for these factors.

Table 1 shows the calculated *K*_d_ values obtained for CAH with the ligands CTZ, DNSA, and SLFA following direct infusion, LESA_premix_, and LESA_ligand_ MS together with those calculated from SPR measurements and literature values. (Examples of SPR sensograms for each ligand are given in Figure S6, Supporting Information, along with the derived binding parameters; see Table S1, Supporting Information.) The *K*_d_ values calculated for LESA_premix_ and LESA_ligand_ MS are given with (i) *P*_0_ and *L*_0_ corrected for the LESA extraction efficiency and dilution, (ii) only *P*_0_ corrected for the LESA extraction efficiency and dilution, and (iii) uncorrected *P*_0_ and *L*_0_.

**Table 1:** Summary of the Experimentally-Derived *K*_d_ Values, and Comparison with Literature Values*

ligand	direct infusion mean *K*_d_ ± STD (μM)	LESA_premix_ mean *K*_d_ ± STD (μM)	LESA_ligand_ mean *K*_d_ ± STD (μM)	SPR mean *K*_d_ ± STD (μM)	literature mean *K*_d_ ± STD (μM)
CTZ	0.28 ± 0.56	0.21 ± 0.14^a^	0.60 ± 0.52^b^	0.28 ± 0.01	2.63 ± 0.14, MS, bovine CAHII^48^
		0.26 ± 0.53^c^	0.98 ± 0.70^a^		0.06 ± 0.02, MS, bovine CAHII^9^
			0.19 ± 0.66^c^		
DNSA	12.49 ± 1.69	11.06 ± 6.75^a^	0.37 ± 0.29^b^	1.14 ± 0.32	0.34 ± 0.04, SPR, 25 °C, bovine CAHII;^33^ 0.36 ± 0.04, ITC, 25 °C, bovine CAHI;^33^ 0.42 ± 0.10, stopped-flow fluorescence, 25 °C, bovine CAHII^33^
		29.93 ± 23.31^c^	0.43 ± 0.31^a^		0.46 ± 0.01, fluorescence, human CAI;^49^ 2.74 ± 0.08, fluorescence, human CAHII;^49^ 0.84, fluorescence, bovine CAHII^49^
			0.37 ± 1.18^c^		0.45 ± 0.10, back scattering interferometry, bovine CAHII^50^
					0.44 ± 0.12, SPR, 25 °C, bovine CAHII^50,51^
SLFA	3.02 ± 1.68	1.73 ± 0.55^a^	1.66 ± 0.89^b^	4.42 ± 1.39	63.5 ± 15.8, colorim. titration, 23 °C;^35^ 89.7 ± 21.7, 37 °C, human CAHI^35^
		1.89 ± 1.45^c^	1.63 ± 0.88^a^		14.9 ± 3.8, colorim. titration, 23 °C, human CAHII;^35^ 28.1 ± 6.0, colorim. titration, 37 °C, human CAHII^35^
			0.22 ± 0.58^c^		0.924, SPR, 25 °C, human CAHI;^27^ 145.7 ± 10, MS, human CAHI^27^
					125.5 ± 18.7, ITC, 25 °C, human CAHI^27^
					0.57 ± 0.09, back scattering interferometry, bovine CAHII^50^
					3.1 ± 1.1, SPR, 25 °C, bovine CAHII^50,51^

* Literature reference values are displayed with an indication of CAH isoform, temperature, and analytical technique if provided. *K*_d_ values determined from LESA experiments are shown before and after corrections of *L*_0_ and/or *P*_0_.

a *P*_0_ and *L*_0_ corrected for LESA extraction efficiency and dilution.

b *P*_0_ corrected for extraction efficiency and dilution.

c Uncorrected for LESA extraction efficiency and dilution.

For CAH and CTZ, there is good agreement between the *K*_d_ values calculated from direct infusion MS, *P*_0_- and *L*_0_-corrected LESA_premix_ MS, and SPR measurements. The measured values fall within the range described in the literature. For CAH and DNSA, there is good agreement between the *K*_d_ values measured by direct infusion and *P*_0_- and *L*_0_-corrected LESA_premix_ MS. For CAH and SLFA, both the uncorrected and the corrected *K*_d_ values calculated from LESA_premix_ MS are in reasonable agreement with those calculated by direct infusion MS. Differences between the reported *K*_d_ values from the SPR and MS measurements have been noted previously and the limitations discussed.^27^ For the *K*_d_ values calculated from LESA_ligand_ MS measurements, the best agreement with the *K*_d_ calculated from direct infusion MS for CTZ was achieved with the uncorrected value. For DNSA, neither corrected nor uncorrected LESA_ligand_ values were in agreement with that obtained following direct infusion MS, although all were closer to those obtained by SPR. For SLFA, corrected values were in the best agreement with that obtained by direct infusion.

### Calculation of *K*_d_ Values Following Direct Infusion and LESA_premix_ MS

Conventionally, the *K*_d_ values are calculated from the direct infusion MS measurements after careful tuning, assuming that no major ligand dissociations occur in source or during detection and that the stoichiometry of a given protein–ligand (PL) complex formed in solution is maintained in the gas phase;^12^ however, improved accuracy in the *K*_d_ values has been demonstrated when in-source dissociation is taken into account.^24,25^ During LESA, the air-dried protein and/or protein–ligand spots are resolubilized and extracted from the substrate for MS. The extraction efficiency is below 100% if not all of the protein or ligand deposited is successfully extracted into the LESA droplet. Moreover, in the extraction process, the dried droplet is extracted into a larger volume (1.5 μL deposited sample volume extracted in 5 μL, i.e., ∼3.33-fold dilution). To address these factors, the extraction efficiencies of the protein and free ligand were determined, enabling us to calculate the corrected *P*_0_ and *L*_0_ values which were then used to determine the *K*_d_ values using eq 1.

First, we assumed that the extraction efficiencies of the free, unbound protein and the ligand-bound protein were the same. To account for the combined dilution and extraction efficiency effects for the protein, we deposited and extracted CAH mimicking the LESA_premix_ conditions (Figure S7, Supporting Information) but collected the extracted sample rather than introducing it to the mass spectrometer. The protein concentration was determined by absorbance at 280 nm. An example of a representative absorption spectrum is shown in Figure S8, Supporting Information, for 10 μM CAH and 10 μM CAH following LESA_premix_ sampling. The mean concentration (*n* = 15) after LESA_premix_ sampling of 10 μM CAH protein spots was 1.51 ± 0.59 μM (Tables S2a and S2b, Supporting Information), indicating that the extraction efficiency was approximately 50%. These data also demonstrate the variation in protein sampling by LESA which can arise due to variable spreading of the sampling droplet, and therefore area sampled, and variation in the proportion of sampling solvent recovered on reaspiration.^52^ The corrected protein concentration was used in subsequent *K*_d_ value calculations for the LESA_premix_ conditions.

To estimate a correction factor for the combined effects of the LESA_premix_-associated dilution and extraction efficiency for each ligand, we conducted LESA extraction, sample collection, and UV–vis spectrophotometry of the three compounds at various concentrations. A range of concentrations for each ligand without LESA sampling served as reference values. For all three ligands, the wavelength with maximum absorbance (λ_max_) was determined experimentally (see Figure S9, Supporting Information, λ_max_ = 277 (CTZ), 244 (DNSA), and 258 nm (SLFA)) The absorbance at λ_max_ was plotted versus the ligand concentration, where it was evident that the mean absorbance values of the reference data points were higher than those following LESA_premix_ sampling. A correction factor was calculated for each ligand across all points based on the ratio of the absorbance of the solution reference sample and the equivalent LESA sample. The mean correction factor was determined to be 0.32 ± 0.03 for CTZ, 0.33 ± 0.05 for DNSA, and 0.39 ± 0.08 for SLFA (Tables S3–S5, Supporting Information), indicating the extraction efficiencies were ∼100% for all concentrations after accounting for the dilution upon LESA_premix_ sampling. These mean correction factors ± 1 STD were used to determine adjusted *L*_0_ values for the LESA_premix_ conditions.

The data used for *K*_d_ value determination by direct infusion and LESA_premix_ are shown in Figure S10, Supporting Information. The results are summarized in Table 1. For CTZ and SLFA, the corrected and uncorrected *K*_d_ values do not differ significantly. For DNSA, however, the uncorrected *K*_d_ value is over 2-fold greater than that for direct infusion but correction of *P*_0_ and *L*_0_ results in good agreement between the *K*_d_ values determined by direct infusion MS and LESA_premix_ MS.

### Calculation of *K*_d_ Values Following LESA_ligand_ MS

Our initial assumption for the LESA_ligand_ mode was that only *P*_0_ would require correction for LESA extraction efficiency and dilution, as the ligand is in the LESA solvent. We determined the corrected *P*_0_ concentration by mimicking the LESA_ligand_ sampling and collecting the sample for UV–vis spectrophotometry. A representative UV–vis spectrum is shown in Figure S11, Supporting Information. The mean extracted protein concentration (*n* = 10) was calculated to be 2.60 ± 0.62 μM (Table S6, Supporting Information). This value was used in subsequent *K*_d_ value calculations as the corrected *P*_0_. Notably, this value is ∼2-fold higher than the value obtained for LESA_premix_, likely due to the repeated mix steps in this LESA mode. (In addition, we could not rule out some solvent evaporation during the extended length of sampling, although the sample stage was kept covered at a constant 21 °C.) Individual titration plots used for determination of *K*_d_ values, with corrected *P*_0_, can be found in Figure S12, Supporting Information.

The general lack of agreement between *K*_d_ values determined by direct infusion MS and LESA_ligand_ MS despite correction of *P*_0_ suggested that our assumption about *L*_0_ was incorrect. We next considered whether the apparent *L*_0_ after LESA_ligand_ may increase as a consequence of evaporation during the 10 min sampling process. The sampling process, in which ligand solutions comprising a range of concentrations were deposited, aspirated, mixed, and reaspirated from the foil-covered slide, was recreated, and the resulting samples were collected. The absorbance spectra of these samples were compared with reference solutions that had not been subjected to any manipulation (Figure S13, Supporting Information). For DNSA and SLFA, the pairwise comparison of absorbances at the maximum wavelength (Figures S13f and S13i, Supporting Information) revealed that at higher concentrations *L*_0_ was higher following LESA_ligand_ sampling than that for the reference solution. Nevertheless, the mean correction factor across the concentration range was ∼1 in both cases (1.063 and 0.9904 for DNSA and SLFA, respectively, see Tables S7 and S8, Supporting Information). For CTZ, the pairwise comparison revealed higher absorbances for the LESA_ligand_ samples at all concentrations (Figure S13c, Supporting Information). Moreover, a blue shift in the absorption spectrum was observed. The calculated mean correction factor was 1.5-fold higher than the initial ligand concentrations (Table S9, Supporting Information). The *K*_d_ values were calculated using corrected values for both *P*_0_ and *L*_0_ (see Table 1 and Figure S14, Supporting Information); however, the fits and agreement between values from direct infusion and LESA_premix_ did not improve. For completeness, we also calculated the *K*_d_ values with no correction for *P*_0_ and *L*_0_ (Figure S14, Supporting Information). For CTZ, the uncorrected *K*_d_ value is in best agreement with the value obtained by direct infusion. For DNSA, correction of *L*_0_ and/or *P*_0_ did not significantly change the calculated *K*_d_ values. For SLFA, the corrected values were in better agreement than the uncorrected values. It is worth noting that the binding kinetics may also result in differences in the *K*_d_ values determined via LESA_premix_ and LESA_ligand_. For the protein–ligand interactions studied here, the calculated time to 99% occupancy was less than 10 min in all cases. For protein–ligand interactions with slower kinetics, it may be necessary to increase the incubation times (LESA_premix_) or number of mix and repeat samples (LESA_ligand_).

### Structural Insights

We also considered whether the discrepancies seen for LESA_ligand_ sampling might be due to conformational changes induced during the drying of the protein spots, that is, if structural changes and additional conformers are produced on drying which are maintained following LESA extraction then there is the possibility that a population of protein exists that is unable to bind ligand. To test that, we coupled native LESA MS with traveling wave ion mobility spectrometry for a single ligand concentration (30 μM) for each compound in the presence of controls. All mass spectra (Figure S15, Supporting Information) displayed a narrow charge state distribution from 8^+^ to 11^+^. The 8^+^ ions showed multiple conformers (Figure S16, Supporting Information); however, the 8^+^ charge state was not observed in high-resolution native MS and was not part of the titration plots described above. For the 9^+^ charge state, two conformations were observed for CAH following LESA whereas a single peak was observed following direct infusion, as observed previously.^53^ It is also possible that the 9^+^ ion shows some heterogeneity in the gas phase, supported by the observation of two conformers with different CCSs reported by Harrison et al.^54^ The drift time profiles for the ligand-bound CAH suggest binding to both the major and the minor conformers. The 10^+^ and 11^+^ ions were characterized by one main, dominant conformation in both direct infusion and LESA modes.

Table S10, Supporting Information, summarizes the estimated mean ^TW^CCSs_N2→He_ of the ligand-bound and free charge states and the CCSs of CAH only. Calculation of the ^TW^CCSs_N2→He_ in the presence of the three CAH inhibitors indicates that the coordination of these ligands in the active center of CAH do not result in a significant increase in ^TW^CCSs_N2→He_. Experimental CCS values yielded similar values for direct infusion, LESA_premix_, and LESA_ligand_ samples.

Finally, we assessed the near-UV secondary structure of CAH in the presence and absence of CTZ, DNSA, and SLFA in a range of concentrations. The circular dichroism spectra did not reveal any major conformational rearrangements in the secondary structure of the protein upon interactions with these ligands (Figure S17, Supporting Information).

## Conclusions

The results show that native LESA MS is a potentially useful tool for quantitative determination of protein–ligand interactions using CAH and three inhibitors covering submicromolar to low micromolar *K*_d_ values as examples of monovalent interactions. To estimate the broader utility of LESA-MS, it would be particularly useful to examine a variety of larger proteins including those with a number of subunits capable of binding multiple ligands, proteins with multivalent binding sites, and proteins that bind numerous ligands with positive or negative cooperativity. When using LESA it is necessary to determine corrections for *P*_0_ and *L*_0_ to better describe the binding equilibrium following sampling from a substrate taking into account extraction efficiency and dilution effects. We found a good match between the *K*_d_ values derived from data obtained following direct infusion MS and LESA_premix_ MS for all three ligands. In contrast, the *K*_d_ values from the LESA_ligand_ sampling were inconsistent, and this could not be addressed by the corrections described here. We found that although LESA_ligand_ MS is not suitable for accurate *K*_d_ value calculations, it can still confirm whether a ligand can bind or not, and CCS measurements can be observed with comparable values to those from direct infusion MS and LESA_premix_ MS. Our findings suggest that native LESA MS has the potential to be developed as an automated, high-throughput platform to support drug development.

## Supporting Information Available

Extended methods, software for calculation of AUCs and fitting of titration curves, chemical structures of ligands, mass spectra, SPR sensograms and calculations, photographs of the advanced user interface, UV absorption spectra, calculation of corrected *P*_0_ and *L*_0_ values, titration plots, TWIMS mass spectra and drift time profiles, calculated collision cross sections, CD spectra (PDF)
Matlab AUC calculations; Python KD fitting (ZIP)
