# Supplementary material for: Quantitative Characterization of Three Carbonic Anhydrase Inhibitors by LESA Mass Spectrometry
Source: J Am Soc Mass Spectrom. 2022 Jun 8;33(7):1168–75. doi: 10.1021/jasms.2c00024 (PMC9264382; doi:10.1021/jasms.2c00024)
Supplement: Supplementary file 1 — js2c00024_si_001.pdf [file js2c00024_si_001.pdf]

Supporting Information

Quantitative characterisation of three carbonic anhydrase inhibitors by LESA mass spectrometry

Eva Illes-Toth[1], Christopher J. Stubbs[2], Emma K. Sisley[1], Jeddidiah Bellamy-Carter[1], Anna L. Simmonds[1], Todd H. Mize[1], Iain B. Styles[3], Richard J. A. Goodwin[4], Helen J. Cooper[1*]

1. School of Biosciences, University of Birmingham, Birmingham, B15 2TT, UK

2. Mechanistic and Structural Biology, Discovery Sciences, R&D, AstraZeneca, Cambridge, CB4 0WG, UK

3. School of Computer Science, University of Birmingham, Birmingham, B15 2TT, UK; The Alan Turing Institute, London, NW1 2DB, UK; Centre of Membrane Proteins and Receptors (COMPARE), University of Birmingham, B15 2TT, and University of Nottingham, NG7 2RD, Midlands, UK

4. Imaging and Data Analytics, Clinical Pharmacology & Safety Sciences, BioPharmaceuticals R&D, AstraZeneca, Cambridge, CB4 0WG, UK

* To whom correspondence should be addressed: h.j.cooper@bham.ac.uk

Table of Contents

Supplemental File 1: Details for peak area calculation and titration curve fitting and associated .m files and jupyter notebook.
Extended Methods
Fig S1. Chemical structure of CTZ, DNSA and SLFA
Fig S2. Deconvoluted mass of CAH
Fig S3. Mass spectra of CAH in the presence CTZ obtained in direct infusion mode, following $LESA_{premix}$ and $LESA_{ligand}$ sampling
Fig S4. Mass spectra of CAH in the presence DNSA obtained in direct infusion mode, following $LESA_{premix}$ and $LESA_{ligand}$ sampling
Fig S5. Mass spectra of CAH in the presence SLFA obtained in direct infusion mode, following $LESA_{premix}$ and $LESA_{ligand}$ sampling
Fig S6. Sensograms for kinetic analysis of CAH with various concentrations of CTZ, DNSA and SLFA
Table S1. Different kinetic parameters of SPR
Fig S7. The Advanced User Interface (AUI)
Fig S8. UV-vis absorption spectra of CAH
Table S2 a and **b**. Calculation of a corrected P_0 for extracted CAH concentration after $LESA_{premix}$
Fig S9. Absorbances for corrected L_0 following $LESA_{premix}$ for CTZ, DNSA and SLFA
Table S3 a and **b**. Calculation of corrected L_0 for CTZ following AUI sampling closely resembling $LESA_{premix}$
Table S4 a and **b**. Calculation of corrected L_0 for DNSA following AUI sampling closely resembling $LESA_{premix}$
Table S5 a and **b**. Calculation of corrected L_0 for SLFA following AUI sampling closely resembling $LESA_{premix}$
Fig S10. Titration plots of CTZ, DNSA and SLFA following $LESA_{premix}$ with no corrections for P_0 and L0
Fig S11. UV absorption spectrum of CAH sampled with AUI closely matching $LESA_{ligand}$ conditions
Table S6 a and **b**. Calculation of a corrected P_0 for extracted CAH concentration matching $LESA_{ligand}$
Fig S12. Titration plots of CTZ, DNSA and SLFA following $LESA_{ligand}$ with corrections for P_0 only
Fig S13. Absorbances for corrected L_0 following $LESA_{ligand}$ for CTZ, DNSA and SLFA
Table S7 a and **b**. Calculation of corrected L_0 for DNSA following AUI sampling closely resembling $LESA_{ligand}$
Table S8 a and **b**. Calculation of corrected L_0 for SLFA following AUI sampling closely matching $LESA_{ligand}$
Table S9 a and **b**. Calculation of corrected L_0 for CTZ following AUI sampling closely mimicking $LESA_{ligand}$
Fig S14. Titration plots of CTZ including corrections for both P_0 and L_0 or no corrections at all for $LESA_{ligand}$

Fig S15. TWIMS mass spectra of CAH and its inhibitors obtained in direct infusion mode, following $LESA_{premix}$ and $LESA_{ligand}$

Fig S16. Drift time profiles of CAH and its inhibitors obtained in direct infusion mode, following $LESA_{premix}$ and $LESA_{ligand}$

Table S10. CCS of CAH and its inhibitors obtained in direct infusion mode, following $LESA_{premix}$ and $LESA_{ligand}$

Fig S17. CD spectra of CAH in the absence or presence of CTZ, DNSA and SLFA

Extended Methods

Methods and materials

Sample preparations for titration experiments

Bovine carbonic anhydrase II (CAH) (C5024), chlorothiazide (6-Chloro-2H-1,2,4-benzothiadiazine-7-sulfonamide 1,1-dioxide)(CTZ) (C4911), dansylamide (5-(dimethylamino)-1-naphthalenesulfonamide) (DNSA) (218898) and sulfanilamide (p-Aminobenzenesulfonamide) (SLFA) (S9251) were purchased from Sigma-Aldrich (Dorset, UK). Protein stock solutions of CAH (15 – 25 µM) were prepared by dissolving the lyophilised powder in LC/MS grade water based on its molar extinction coefficient (50,070 M^{-1} cm^{-1}[1, 2]) using a DeNovix DS-II spectrophotometer at 280 nm in protein A_{280} mode. The concentration was adjusted to 10 µM using 25 mM ammonium acetate, pH 7. LC/MS grade solvents were purchased from Fisher Scientific (Loughborough, UK), ammonium acetate was purchased from J. T. Baker (Deventer, The Netherlands). CTZ and SLFA were dissolved at 675 µM concentration in water containing 5 mM NaOH. DNSA was dissolved at 675 µM concentration in 10% (v/v) MeOH and 5 mM NaOH in water. All ligand solutions were further diluted thus the MeOH content was reduced < 0.5% in the case of DNSA and the NaOH content was residual for all. The pH of the solutions introduced for mass spectrometry was checked and found to be neutral.

Direct infusion and LESA sampling ($LESA_{premix}$ and $LESA_{ligand}$)

Samples of 10 µM CAH and 10 µM CAH with varying concentrations of CTZ, DNSA and SLFA were introduced for MS (Orbitrap Elite, Thermo Fisher Scientific, Bremen, Germany) with an Advion TriVersa NanoMate using an electroconductive tip coupled to a chip *in direct infusion mode* from a 96 well microtiter plate for reference. For direct infusion, a 10 µM protein or protein-ligand solution in 25 mM ammonium acetate was introduced directly with the robot was operated with the ChipSoft 8.3.3 software at 1.4 kV capillary voltage, 1.4 - 1.6 mm height and 0.40 psi pressure.

For all LESA analyses, an image of the glass slides covered with Al foil containing the protein and/or protein-ligand sample spots was acquired using an Epson Perfection V300 flatbed photo scanner. The dried droplet locations were selected with the aid of LESA Points software (Advion).

Subsequently, 1.5 µL spots of samples of 10 µM CAH and CAH mixed with different concentrations of CTZ (0.075 µM, 0.1 µM, 1 µM, 2.5 µM, 5 µM, 10 µM, 20 µM and 30 µM) DNSA (1 µM, 1.25 µM, 1.5 µM, 3 µM, 5 µM, 10 µM, 15 µM, 20 µM and 30 µM) and SLFA (2.5 µM, 3.5 µM, 5 µM, 7.5 µM, 10 µM, 15 µM, 20 µM, 25 µM and 30 µM) after 10 min incubation at RT were air-dried and sampled by LESA from the surface of a glass slide at a 60 s dwell time.

$LESA_{premix}$: 5 µL 25 mM NH_4OAc was aspirated from the solvent reservoir, 3 µL was deposited on the surface of the substrate at a 1.4 mm – 1.6 mm height forming a liquid junction, extraction was held at a 60 s dwell time, 3.5 µL was re-aspirated and introduced to the mass spectrometer.

$LESA_{ligand}$: Various concentrations of ligands were placed directly in the extraction solvent. The examined ligand concentrations were 0.05 µM, 0.075 µM, 0.1 µM, 1 µM, 2.5 µM, 5 µM, 10 µM, 20 µM and 30 µM for CTZ, 1 µM, 1.25 µM, 1.5 µM, 2 µM, 3 µM, 5 µM, 10 µM, 15 µM, 20 µM and 30 µM for DNSA and 2.5 µM, 3.5 µM, 5 µM, 7.5 µM, 10 µM, 15 µM, 20 µM, 25 µM and 30 µM for SLFA. Each ligand was used to sample the dried CAH spots in the same manner as above with the exception of including an additional 10 cycle step composed of 1 min repeat-mix steps to mimic the 10 min incubation of step with the temperature control set at 21 °C. This ensured the same length of time of exposure for protein-ligand binding. A baseline

correction was performed by subtracting the absorption of 25 mM ammonium acetate solution sampled the same way.

Travelling wave ion mobility mass spectrometry (TWIMS)

Ubiquitin (U6253), cytochrome c from equine heart (C2506), myoglobin from equine skeletal muscle (M0630) and carbonic anhydrase from bovine erythrocytes (C5024) were purchased from Sigma-Aldrich (Dorset UK) and dissolved at 10 µM concentration based on their molar extinction coefficients (1280 M^{-1} cm^{-1}, 18,600 M^{-1} cm^{-1}, 13,940 M^{-1} cm^{-1} and 50,070 M^{-1} cm^{-1} respectively) at 280 nm in either denaturing buffer (50 % acetonitrile, 1 % formic acid and 49 % water) or in 25 mM aqueous ammonium acetate (J. T. Baker, the Netherlands), pH 7.0. The Synapt G2 S mass spectrometer (Waters, Wilmslow, UK) was operated in positive ion mode at a helium flow rate of 180 mL/min and nitrogen flow rate of 90 mL/min. The settings were: capillary voltage 1.4 - 1.5 kV, cone 50 V, source temperature 80°C, trap collision energy 4 V, transfer collision energy 8 V, trap wave velocity 311 m/s, wave height 6 V, IMS wave velocity 700 m/s, wave heights of 35 V, 36 and 37 V, transfer wave velocity 200 m/s, wave height 4 V, trap bias 45, IMS bias 3 and backing pressure $5.1e^{0}$ mbar. Collision cross section (CCS) calibration was performed measuring drift times of denatured ubiquitin, cytochrome c and apo-myoglobin following protocols as described before[3]. The peak apexes of drift times were taken to calculate CCSs. Reference CCSs were used from the Bush database[4] for ubiquitin and myoglobin and from the Clemmer database[5] for cytochrome c. Experimental CCSs measured in N_2 were expressed as $^{TW}CCS_{N2\rightarrow He}$ using He reference values as part of the calibration procedure. Data were acquired in triplicate in the absence or presence of each ligand, for each acquisition over 200 scans were collected in the 200 - 4000 m/z mass range. Mass calibration was performed externally using CsI clusters in the corresponding mass range. All data were viewed and analysed in MassLynx V4.1 (Waters, Wilmslow, UK) and exported for plotting without any smoothing in Prisms GraphPad 6.01.

Circular dichroism

For assessment of potential secondary structure changes, circular dichroism spectra were obtained between 190 nm to 270 nm on a J-1500 instrument (JASCO Corp, Tokyo, Japan) at scanning speed of 100 nm/min, in continuous mode, with a response time of 1 s, bandwidth of 1 nm, accumulation of 6 spectra, using a cuvette with a path length of 0.1 cm in 25 mM ammonium acetate with or without three different concentrations of CTZ, SLFA and DNSA at 10 µM protein concentration. Ellipticity was expressed as follows: (degcm x 10^{6})/(path length (cm) x conc. (µM) x number of peptide bonds).

Surface Plasmon Resonance

SPR analyses were performed on a Biacore 8K and T200 instruments (Biacore Life Sciences, GE Healthcare, Uppsala, Sweden). CM5 Series S Sensor Chips, N-hydroxysuccinimide (NHS), 1-ethyl-3-(3-dimethylamino-propyl)-carbodiimide (EDC-HCl) and 1 M ethanolamine HCl were obtained from Cytiva. CAH was dissolved to 2 mg/mL in 25 mM ammonium acetate on the day of analysis.

Immobilisation of CAH. The instrument was desorbed and a fresh CM5 sensor chip docked. After priming into running buffer (25 mM ammonium acetate), the CM5 chip was preconditioned with three injections of 50 mM NaOH/1 M NaCl. The sensor surface was activated for 420 s (0.2 M EDC, 0.05 M NHS, 10 µL/min) then 100 µg/mL CAH dissolved in 10 mM sodium acetate pH 5.0 (or running buffer for the reference flow cells) was injected for 300 s. Remaining active esters were blocked with three 60 s injections of 1 M ethanolamine-HCl

pH 8.5. This resulted in ~1000 RU CAH being immobilised on the active flow cells. The immobilisation was performed at 25 °C.

Sample Testing. Samples were tested at 21 °C. Compound stock solutions were prepared at 2.5 mM in 10 mM NaOH. For the titration series, compounds were diluted to 30 μM in running buffer then serially-diluted 2-fold to yield an 11-point dilution series. During each binding cycle, association of ligand with the immobilised protein was monitored for 120 s, and dissociation for 600 s. Each ligand concentration was injected in replicate to obtain corresponding sensograms, n=6 (CTZ) and n=5 (DNSA and SLFA). Double referenced sensograms were analysed in Biacore Insight Evaluation Software (Cytiva) using a kinetic 1:1 binding model yielding the association and dissociation rate constants (k_a and k_d, respectively) allowing calculation of $K_d = k_d/k_a$.

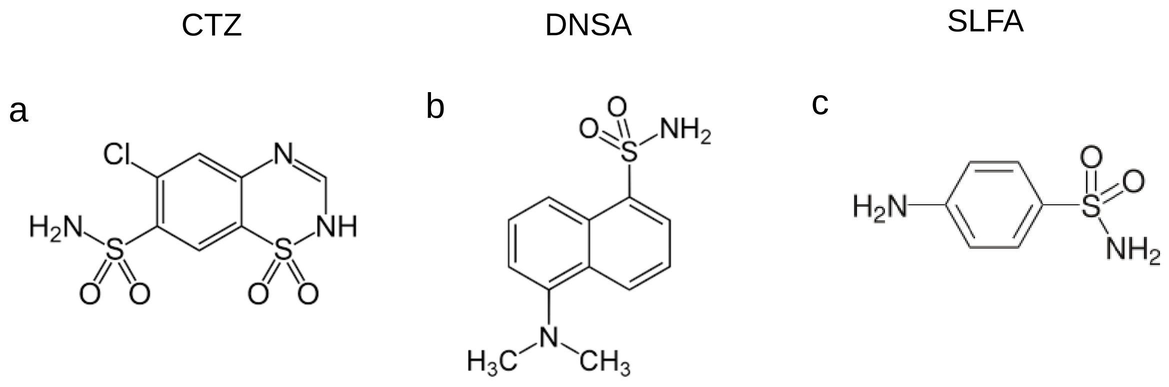

Fig S1. The chemical structure of chlorothiazide (CTZ) (a), dansylamide (DNSA) (**b**) and sulfanilamide (SLFA) (**c**) drawn in PerkinElmer ChemDraw Professional 15.1 (CambridgeSoft Corporation).

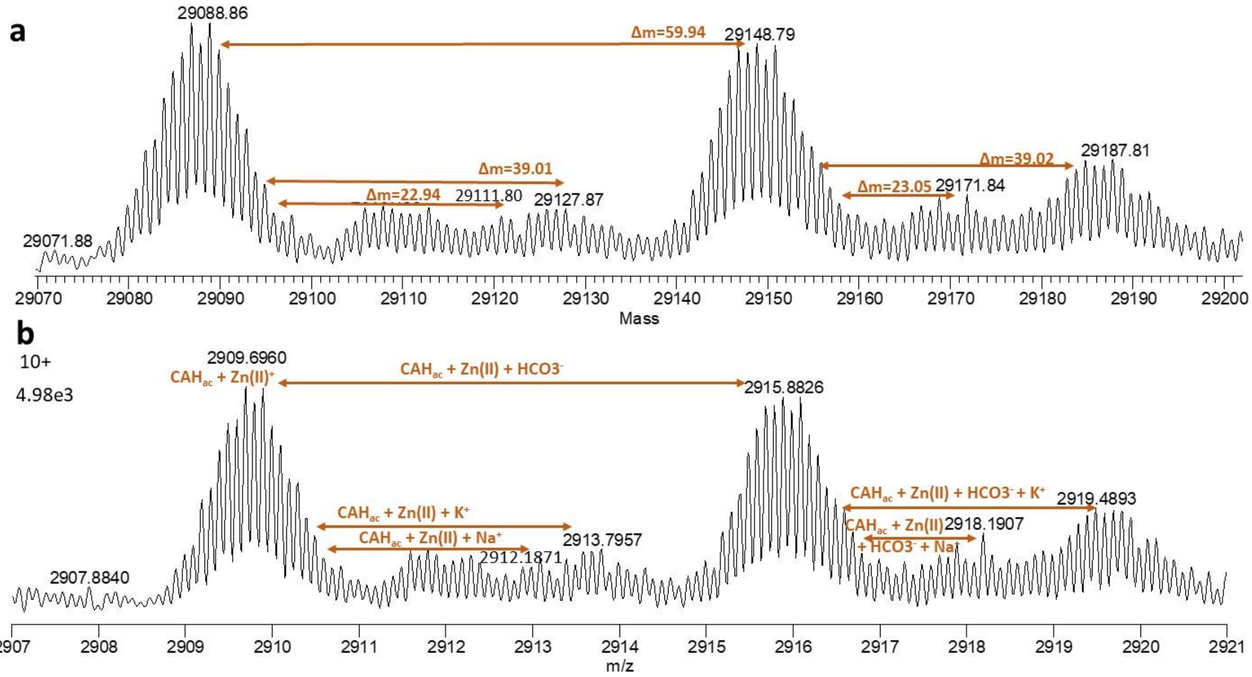

Fig S2. Deconvoluted experimental mass of CAH was found to be 29,088.90 Da using MagTran[6] with the "charge states and then isotopes" option with a S/N set at 2, mass accuracy at 0.5 Da, zero charged mass spectrum (**a**). This agrees well with the theoretical value of 29,088.78 Da (CAH UniProt accession ID: P00921) containing an N-terminal acetylation and addition of a Zn(II) ion. For brevity the acetylated CAH bound to zinc is shortened to P in other figures and that bound to its respective ligand is shortened to PL. The most intense peak after the protonated zinc bound CAH (2918.79 +0.5 Da experimental mass here) has been previously assigned as an acetate adduct[7] or hydrogen carbonate bound ionically to the protein[8]. We followed the latter[8] notation. An expanded region of a high resolution (R: 120, 0000) CAH mass spectrum for the 10+ charge state acquired on a Thermo Orbitrap Elite in positive mode in 25 mM NH_4OAc (**b**).

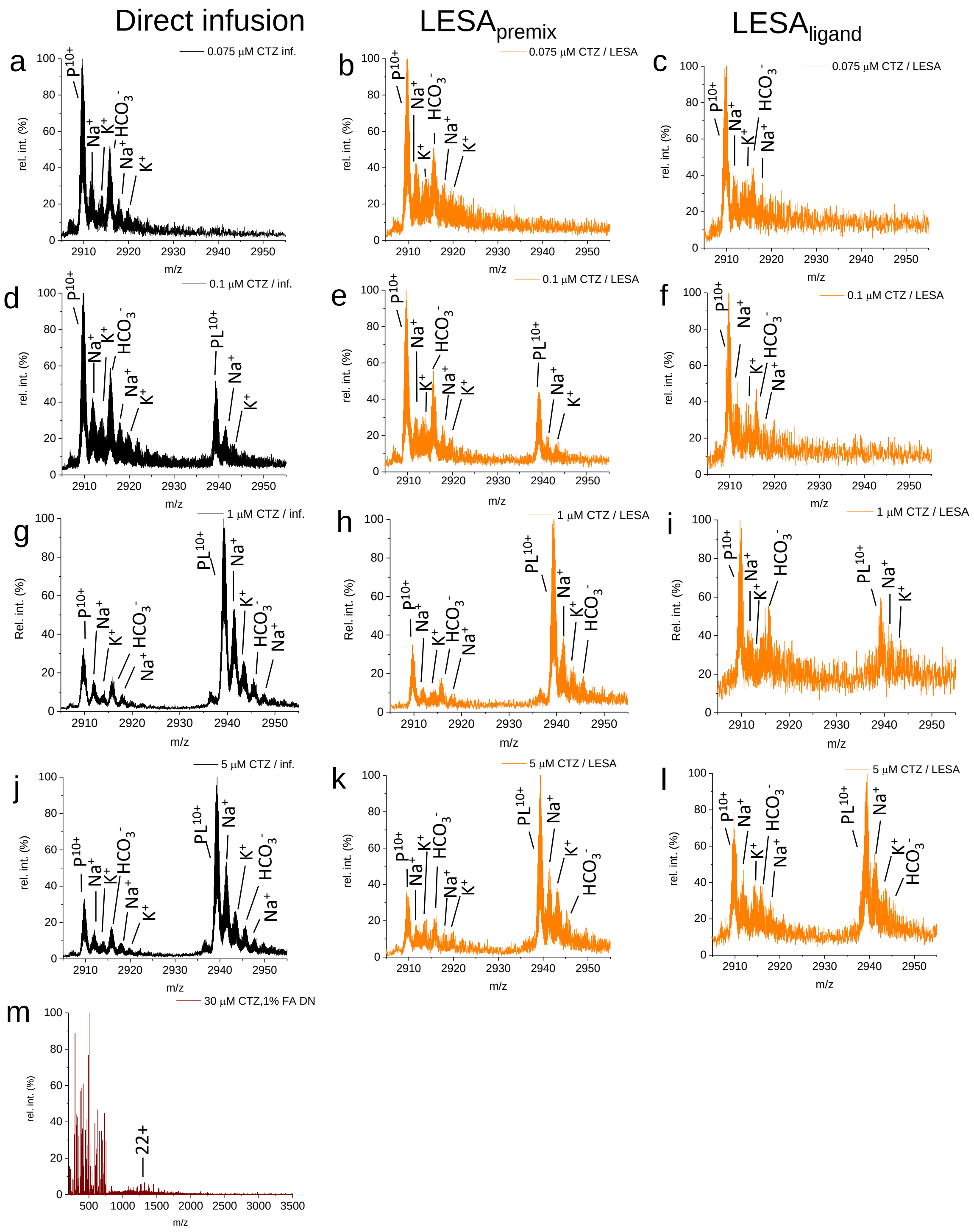

Fig S3. Mass spectra of CAH in the presence of 0.075 µM CTZ obtained in direct infusion mode (**a**), following LESA$_{premix}$ (**b**) and LESA$_{ligand}$ sampling (**c**) at the 10^{+} charge state. Mass spectra of CAH in the presence of 0.1 µM CTZ acquired by direct infusion (**d**), following LESA$_{premix}$ (**e**) and LESA$_{ligand}$ sampling (**f**). Mass spectra of CAH in the presence of 1 µM CTZ obtained in direct infusion mode (**g**), following LESA$_{premix}$ (**h**) and LESA$_{ligand}$ sampling (**i**). Mass spectra of CAH in the presence of 5 µM CTZ following direct infusion (**j**), LESA$_{premix}$ (**k**) and LESA$_{ligand}$ sampling (**l**) for the 10^{+} charge state. All concentrations in the figure legends denote those without any corrections. Addition of 1% FA to 30 µM CTZ and 10 µM CAH led to denaturation of the protein and the loss of ligand binding (**m**). P denotes acetylated CAH bound to zinc. Protein adducts are indicated as Na^{+}, K^{+} and $HCO3^{-}$.

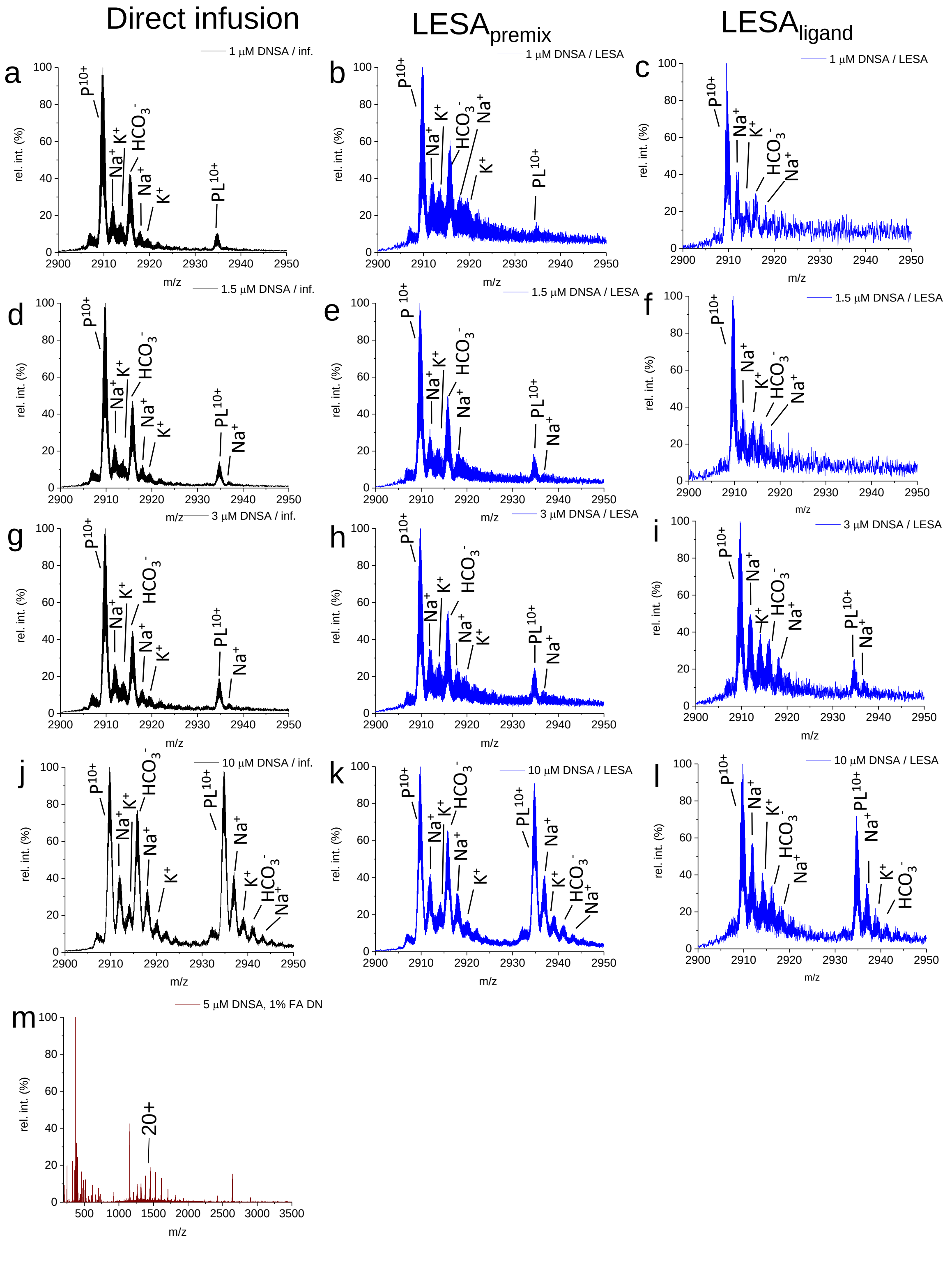

Fig S4. Mass spectra of CAH in the presence of 1 µM DNSA obtained in direct infusion mode (**a**), following LESA$_{premix}$ (**b**) and LESA$_{ligand}$ (**c**) at the 10^{+} charge state. Expanded mass spectra of CAH in the presence of 1.5 µM DNSA acquired by direct infusion (**d**), using LESA$_{premix}$ (**e**) and LESA$_{ligand}$ sampling (**f**). Expanded mass spectra of CAH in the presence of 3 µM DNSA following direct infusion (**g**), LESA$_{premix}$ (**h**) and LESA$_{ligand}$ sampling (**i**). Mass spectra of CAH with 10 µM DNSA following direct infusion (**j**), LESA$_{premix}$ sampling (**k**) and LESA$_{ligand}$ (**l**) for the 10^{+} charge state. The concentrations indicate those without any further corrections. Addition of 1% FA to 10 µM CAH mixed with 5 µM DNSA caused unfolding of the protein and the loss of ligand binding (**m**). P denotes acetylated CAH bound to zinc. Protein adducts are indicated as Na^{+}, K^{+} and HCO3^{-}.

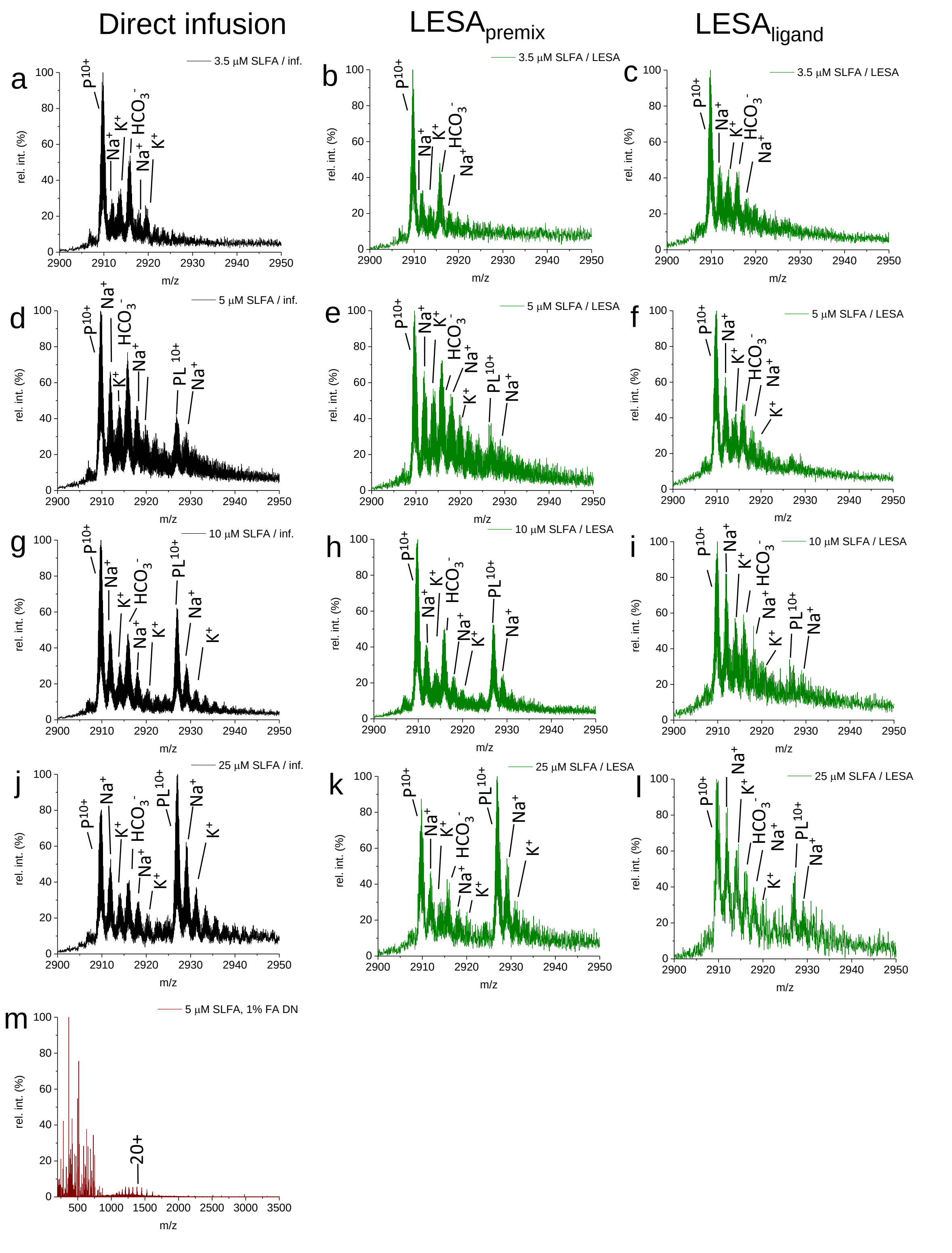

Fig S5. Mass spectra of CAH in the presence of 3.5 µM SLFA obtained by direct infusion (**a**), following $LESA_{premix}$ (**b**) and $LESA_{ligand}$ sampling (**c**) for the 10^{+} charge state. Expanded mass spectra of CAH in the presence of 5 µM SLFA acquired by direct infusion (**d**), using $LESA_{premix}$ (**e**) and $LESA_{ligand}$ (**f**). Mass spectra of CAH in the presence of 10 µM SLFA following direct infusion (**g**), $LESA_{premix}$ (**h**) and $LESA_{ligand}$ sampling (**i**). Mass spectra of CAH in the presence of 25 µM SLFA obtained by direct infusion (**j**), $LESA_{premix}$ (**k**) and $LESA_{ligand}$ sampling (**l**) for the 10^{+} charge state. Ligand concentrations are shown without any corrections. Inclusion of 1% FA to 10 µM CAH with 5 µM SLFA resulted in the unfolding of the protein and the loss of SLFA binding (**m**). P denotes acetylated CAH bound to zinc. Protein adducts are indicated as Na^{+}, K^{+} and $HCO3^{-}$.

SPR. We determined the kinetics of CTZ, DNSA and SLFA binding to CAH across a wide concentration range prepared from serial dilutions up to 30 µM ligand concentration. CAH has been widely used as model system in SPR studies[1, 9-11] and its preparation and handling for immobilisation on biosensor chips is well documented.

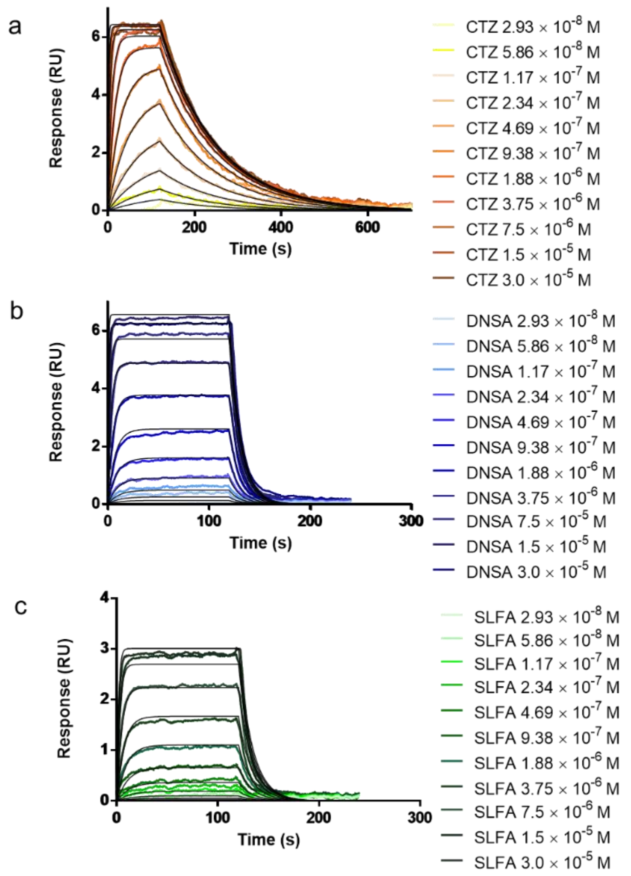

Fig S6. Sensograms for kinetic analysis of CAH with various concentrations of CTZ (a), DNSA (b) and SLFA (c) are shown with global fits of experimental data to a single site model (black continuous lines). All experiments were performed at 21 °C.

Table S1. Arithmetic means ± STD of different kinetic parameters of CAH in the presence of CTZ, DNSA and SLFA determined from SPR measurements; n=5 (DNSA and SLFA) and n=6 (CTZ).

Compound	Mean k_a ± STD (M^{-1} s^{-1})	Mean k_d ± STD (s^{-1})	Mean K_d ± STD (M)
CTZ	2.65E+04 ± 5.78E+02	7.49E-03 ± 3.47E-04	2.83E-07 ± 1.02E-08
DNSA	9.04E+04 ± 3.05E+04	1.03E-01 ± 1.15E-02	1.14E-06 ± 3.20E-07
SLFA	1.93E+04 ± 4.01E+03	8.54E-02 ± 1.01E-02	4.42E-06 ± 1.39E-06

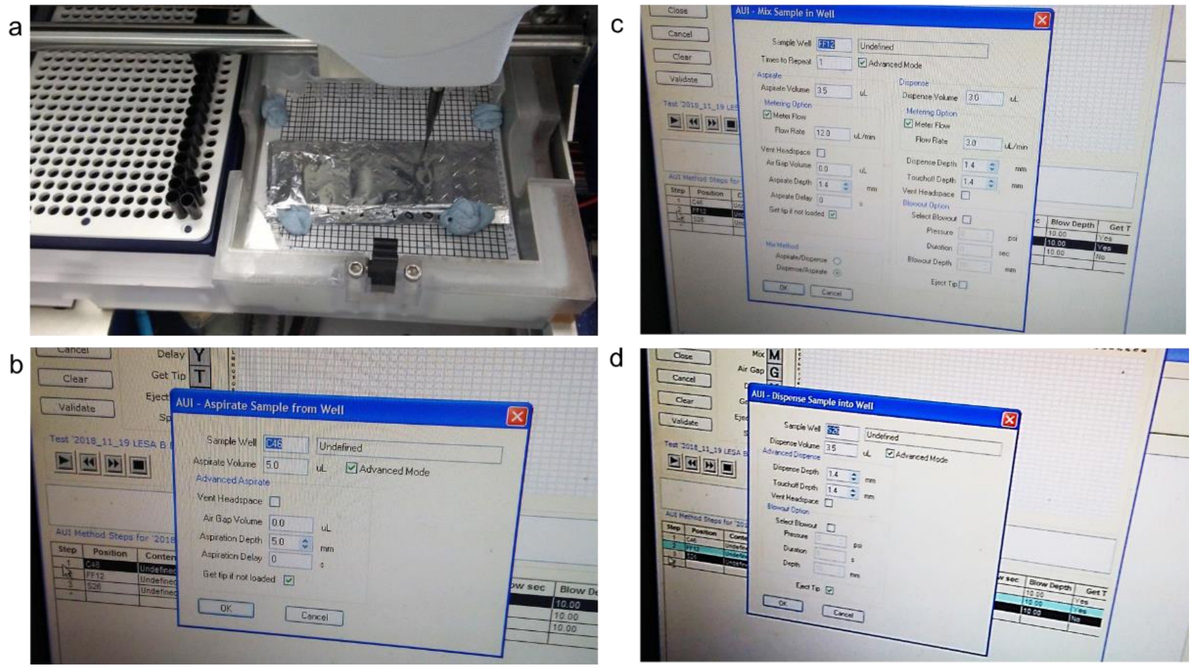

Fig S7. The use of the Advanced User Interface (AUI) mimicking LESA$_{premix}$ conditions. A 1584 well paper grid template is placed under a glass slide covered with foil containing sample spots to guide determination of xy coordinates. Next to the slide a half 96 well microtiter plate is placed that contains the ammonium acetate in a designated well and is used to deposit the extracted aliquots into that subsequently taken for UV-vis spectrophotometry (**a**). The workflow of the AUI is composed of three main stages. The first one being highlighted with black next to the arrow entails aspiration of the ammonium acetates from a defined location of the half 96 well plate (**b**). The next stage includes first the dispensation of solvent to a certain location on the glass slide with a pre-defined xy coordinate at the defined flow rate/time duration, followed by a re-aspiration step that enables collection of the extracted protein (**c**). The aliquot is then dispensed into a specific well in the halved 96 well plate (**d**).

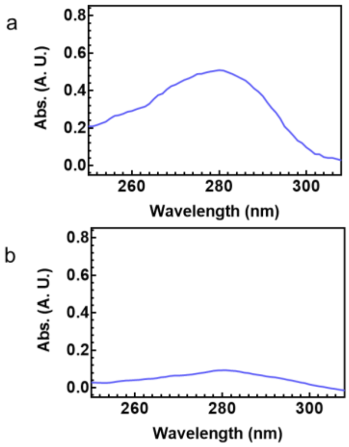

Fig S8. Representative UV absorption spectrum of 10 µM CAH (**a**), 10 µM CAH deposited, air-dried and sampled with AUI closely matching LESA$_{premix}$ conditions (**b**).

Table S2. Calculation of a corrected P_0 based on extracted CAH concentrations using UV absorbance at 280 nm (A_{280}). The absorbance values were converted to concentrations based on the molar extinction coefficient of CAH. From each extracted aliquot two A_{280} readings were taken, n=15 (**a**). The statistical outputs of AUI sampling closely resembling $LESA_{premix}$ conditions calculated in Prism GraphPad 6.01 are shown. The corrected protein value ± one STD was used in the final K_d calculations (**b**).

Table S2 a.

Abs.$_{280\ nm}$ (A.U.)		Concentration (µM)	
0.0749	0.0749	1.495910	1.495910
0.0595	0.0605	1.188340	1.208310
0.0748	0.0665	1.493910	1.328140
0.0492	0.0662	0.982624	1.322150
0.0920	0.0857	1.837430	1.711600
0.0613	0.0726	1.224290	1.449970
0.0714	0.0706	1.426000	1.410030
0.1163	0.1199	2.322750	2.394650
0.0770	0.0891	1.537850	1.779510
0.1011	0.0887	2.019170	1.771520
0.1340	0.1352	2.676250	2.700220
0.0249	0.0230	0.497304	0.459357
0.0603	0.0683	1.204310	1.364090
0.0326	0.0215	0.651088	0.429399
0.0852	0.1096	1.701620	2.188940

Table S2 b.

	Abs.$_{280\ nm}$ (A.U.)	Concentration (µM)
Number of values	15	15
Minimum	0.02395	0.4783
25% Percentile	0.0600	1.198
Median	0.0710	1.418
75% Percentile	0.0949	1.895
Maximum	0.1346	2.688
Mean	0.07556	1.509
Std. Deviation	0.02952	0.5897
Std. Error of Mean	0.007623	0.1522
Lower 95% CI of mean	0.05921	1.183
Upper 95% CI of mean	0.09191	1.836
Sum	1.133	22.64

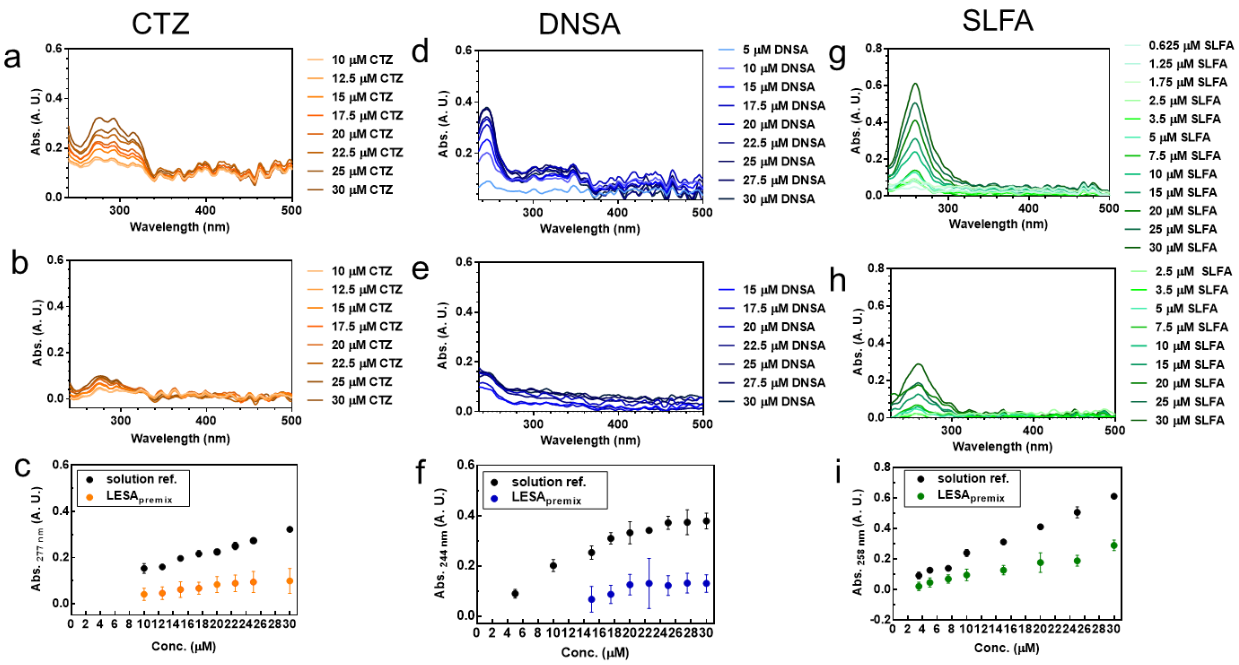

Fig S9. Absorption spectra obtained directly from various CTZ reference solutions (**a**) and absorption spectra of samples following AUI sampling closely matching the $LESA_{premix}$ conditions (**b**). Mean absorbance ± STD of CTZ reference solutions and those sampled by the AUI method at the wavelength of 277 nm (**c**). Absorption spectra measured from various concentration of DNSA working solutions (**d**) and absorption spectra of samples undergone AUI sampling matching the $LESA_{premix}$ conditions (**e**). Mean absorbance ± STD of DNSA reference solutions and those sampled by the AUI method matching the $LESA_{premix}$ conditions at the wavelength of 244 nm (**f**). Absorption spectra attained directly from solutions prepared at different concentrations (**g**) and absorption spectra of SLFA samples following AUI resembling $LESA_{premix}$ conditions (**h**). Mean absorbance ± STD of DNSA reference solutions and those sampled by the AUI method matching $LESA_{premix}$ conditions at the wavelength of 258 nm (**i**). All legends denote the concentration of CTZ, DNSA and SLFA prior to any sample manipulations.

Table S3. Calculation of a correction factor for CTZ following AUI sampling closely resembling $LESA_{premix}$. The absorbance of samples following LESA sampling were compared with absorbance of reference solutions at each different concentration in triplicate; mean values and SEM are given (**a**). The mean value of the correction factor from eight different concentrations is shown with the corresponding ± SEM (**b**) that were used to calculate the mean correction factor ± STD in the final calculations respectively.

Table S3 a.

	Corr. = $LESA_{premix}$ / ref. at $Abs._{277\ nm}$	
Concentration (μM)	Mean	SEM
10.000	0.2663755	0.07227074
12.500	0.2855648	0.07018127
15.000	0.3129252	0.07141756
17.500	0.307396	0.05083121
20.000	0.3692423	0.06249592
22.500	0.3526667	0.06053115
25.000	0.3437119	0.06789904
30.000	0.3062176	0.06853145

Table S3 b.

Mean	0.3180
STD	0.03481
SEM	0.01231

Table S4. Calculation of a correction factor for DNSA following sampling with AUI closely resembling $LESA_{premix}$ conditions. The absorbance of samples following AUI sampling closely matching $LESA_{premix}$ were compared with absorbance of reference solutions at each different concentration (triplicate measurements); mean values and SEM are given (**a**). The mean correction factor value from seven different concentrations is shown with the corresponding SEM (**b**) that were used to calculate the mean correction factor ± STD.

Table S4 a.

	Corr. = $LESA_{premix}$ / ref.at Abs.$_{244\ nm}$	
Concentration (µM)	Mean	SEM
15.000	0.2648221	0.08390142
17.500	0.2798708	0.0493652
20.000	0.37751	0.05786061
22.500	0.3813477	0.1193145
25.000	0.3278027	0.0449839
27.500	0.3519179	0.05077823
30.000	0.34375	0.04094474

Table S4 b.

Mean	0.3324
STD	0.04525
SEM	0.01710

Table S5. Calculation of a correction factor for SLFA following sampling with the use of AUI closely resembling $LESA_{premix}$. The absorbance of LESA samples were compared with absorbance of reference solutions at each different concentration (triplicate measurements); mean values and SEM are given (**a**). The mean correction factor value from eight different concentrations is shown ± SEM (**b**) that were used to calculate the mean correction factor for L_0 concentration ± STD.

Table S5 a.

	Corr. = $LESA_{premix}$ / ref. at Abs.$_{258\ nm}$	
Concentration (µM)	Mean	SEM
3.500	0.2233577	0.1264563
5.000	0.3578948	0.09808842
7.500	0.4928058	0.08278406
10.000	0.3960307	0.06693573
15.000	0.4066989	0.04114911
20.000	0.428815	0.06381217
25.000	0.3719657	0.0324859
30.000	0.4733624	0.02326547

Table S5 b.

Mean	0.3939
STD	0.08311
SEM	0.02938

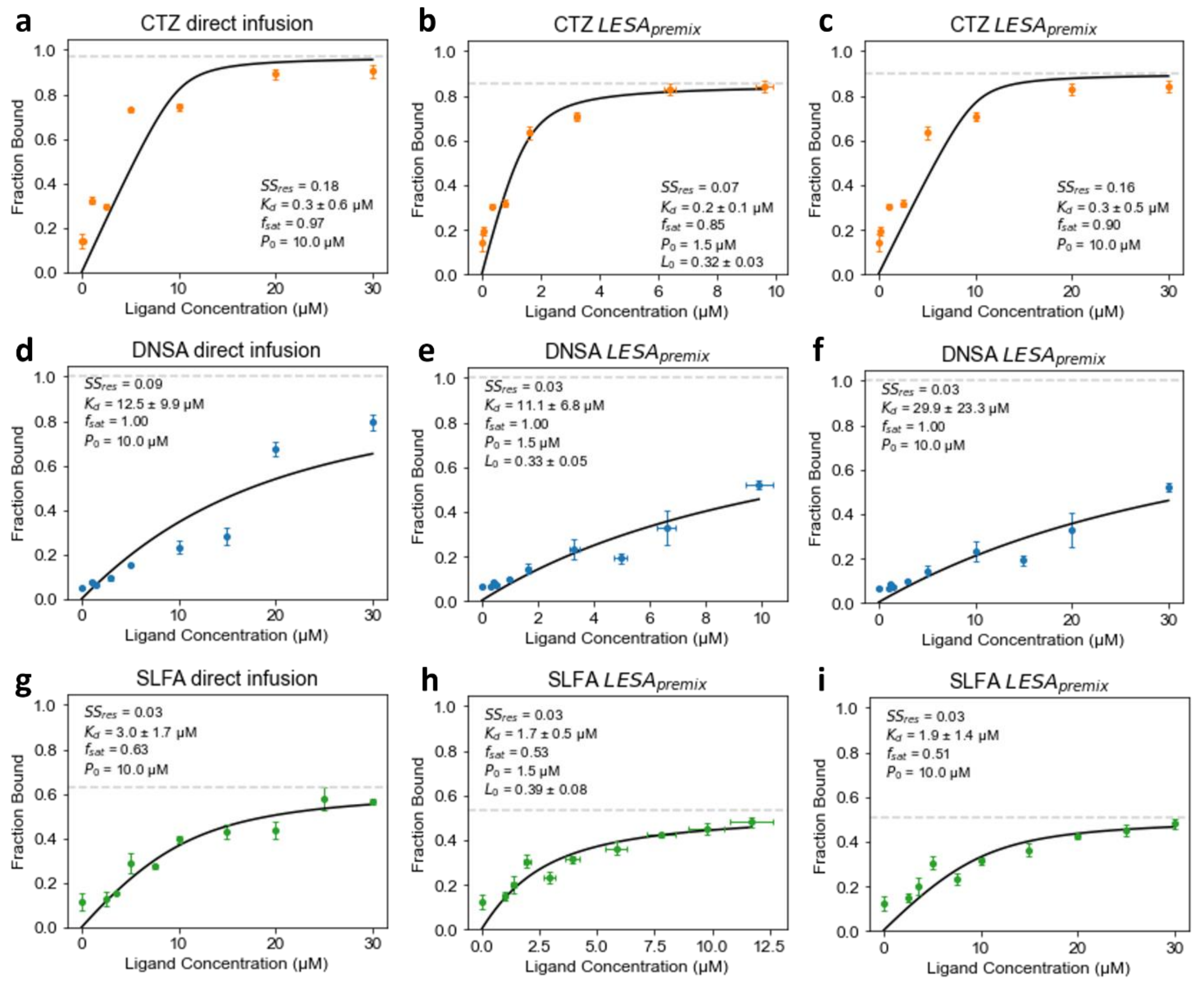

Fig S10. Titration plots following direct infusion and $LESA_{premix}$ sampling with corresponding K_d values ± STD. Titration plots of CTZ obtained in direct infusion mode (**a**), following $LESA_{premix}$ with P_0 and L_0 corrections shown in the bottom right (**b**) and following $LESA_{premix}$ with no corrections applied (**c**). DNSA titration plots following direct infusion (**d**) and $LESA_{premix}$ with P_0 and L_0 corrections indicated (**e**) and following $LESA_{premix}$ with uncorrected P_0 and L_0 (**f**). Titration plots of SLFA following direct infusion (**g**) and following $LESA_{premix}$ with corrected P_0 and L_0 displayed in the bottom right (**h**) and following $LESA_{premix}$ without any corrections (**i**). Fraction bound (Fr. Bound) is plotted vs. initial L_0 concentrations (Conc. (μM). All data points show mean values ± STD for including all three charge states of CAH acquired following $LESA_{premix}$ sampling, n=3; the black lines show the fit used for determination of K_d values. SS_{res} is the measure of the goodness of fitting. f_{sat} for each fit is displayed in each plot.

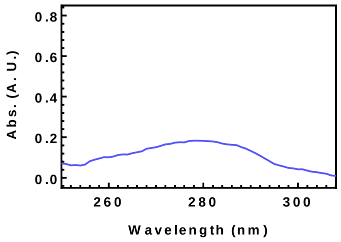

Fig S11. Representative UV absorption spectrum of 10 µM CAH deposited, air-dried and sampled with AUI closely matching $LESA_{ligand}$ conditions.

Table S6. Calculation of a corrected P_0 based on extracted CAH concentrations using UV absorbance at 280 nm (A_{280}) for $LESA_{ligand}$. The absorbance values were converted to concentrations using the molar extinction coefficient of CAH. From each extracted aliquot two A_{280} readings were taken, n=10 (**a**). The statistical outputs of AUI sampling mimicking $LESA_{ligand}$ conditions calculated in Prism GraphPad 6.01. The mean concentration value (µM) was used for correction of P_0 in the final K_d calculations (**b**).

Table S6 a.

$Abs._{280\ nm}$ (A.U.)		Concentration (µM)	
0.0965	0.1116	1.9273	2.2289
0.1220	0.1147	2.4366	2.2908
0.1528	0.1420	3.0517	2.8360
0.0910	0.0890	1.8175	1.7775
0.1405	0.1307	2.8061	2.6104
0.1082	0.1300	2.1610	2.5964
0.1811	0.1652	3.6169	3.2994
0.1278	0.1307	2.5524	2.6104
0.1718	0.1815	3.4312	3.6249
0.0933	0.0792	1.8634	1.5818

Table S6 b.

	$Abs._{280\ nm}$ (A.U.)	Concentration (µM)
Number of values	10	10
Minimum	0.08625	1.723
25% Percentile	0.1005	2.008
Median	0.1242	2.480
75% Percentile	0.1538	3.072
Maximum	0.1767	3.528
Mean	0.1280	2.556
STD	0.03122	0.6236
SEM	0.009874	0.1972
Lower 95% CI of mean	0.1056	2.110
Upper 95% CI of mean	0.1503	3.002
Sum	1.280	25.56

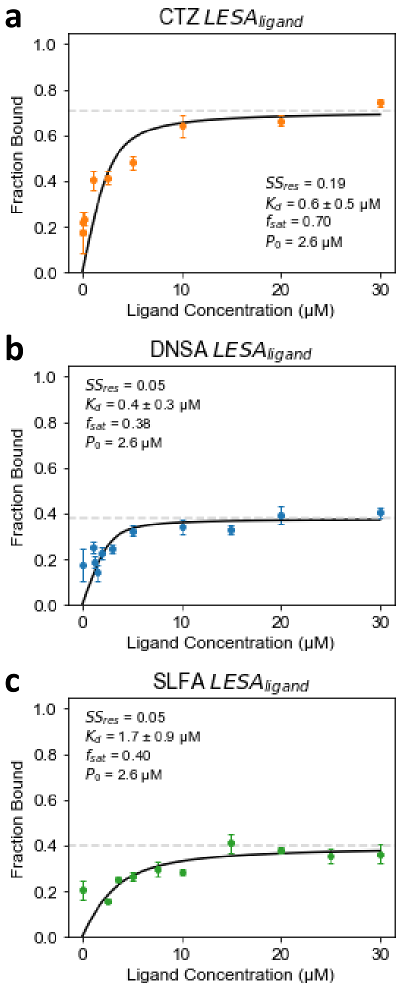

Fig S12. Titration plots following $LESA_{ligand}$ for CTZ (**a**), for DNSA (**b**) and for SLFA (**c**). In each case, a correction was applied for P_0 only to determine the K_d ± STD. Fraction bound is plotted vs. initial L_0 concentrations (Conc. (μM)) Data points represent mean values with one STD for all three charge states of CAH acquired following $LESA_{ligand}$ sampling, n=3; the black lines show the fit used for determination of K_d values. SS_{res} indicates the goodness of fit. f_{sat} is shown for each plot.

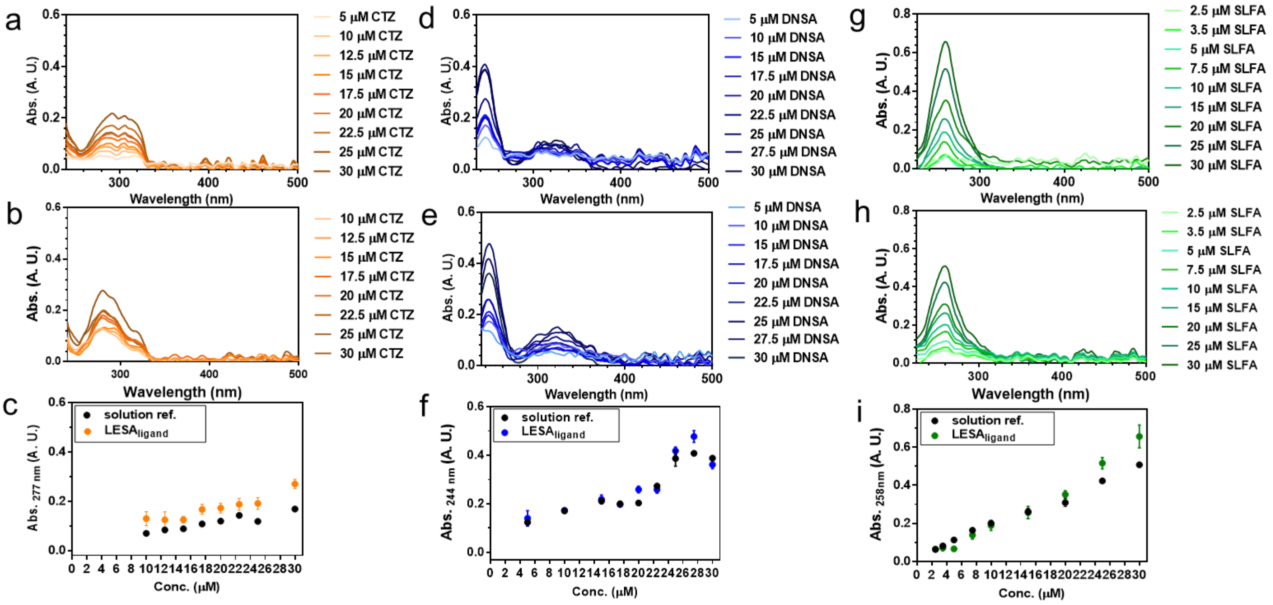

Fig S13. Absorption spectra of CTZ reference solutions at various concentrations (**a**) and absorption spectra of samples undergone AUI sampling closely matching the $LESA_{ligand}$ conditions. The legends here indicate the original ligand concentrations without any further corrections (**b**). Mean absorbance ± STD of CTZ reference solutions and those sampled by the AUI method mimicking $LESA_{ligand}$ sampling at the wavelength of 277 nm (**c**). Absorption spectra of reference DNSA solutions (**d**), and samples undergone AUI sampling matching the $LESA_{ligand}$ conditions (**e**). Mean absorbance ± STD of DNSA reference solutions and those sampled by the AUI method resembling the $LESA_{ligand}$ conditions at the wavelength of 244 nm (**f**). Absorption spectra measured directly from solutions prepared at different SLFA concentrations (**g**), and absorption spectra of SLFA samples following AUI resembling $LESA_{ligand}$ conditions (**h**). Mean absorbance ± STD of DNSA reference solutions and those sampled by the AUI method matching $LESA_{ligand}$ conditions at the wavelength of 258 nm (**i**). The legends show concentrations of CTZ, DNSA and SLFA without any corrections applied.

Table S7. Calculation of a correction factor for DNSA following AUI sampling closely matching $LESA_{ligand}$ sampling. The absorbance of samples following AUI sampling resembling $LESA_{ligand}$ sampling were compared with the absorbance of reference solutions at each different concentration (triplicate measurements); mean values and SEM are given (**a**). The mean correction factor value from nine different concentrations is shown with the corresponding one STD and SEM (**b**) that were used as the correction factor ± STD for L_0 of DNSA in an attempt for calculating K_d respectively.

Table S7 a.

	Corr. = $LESA_{ligand}$ / ref. at $Abs._{244nm}$	
Concentration (μM)	Mean	SEM
5.000	1.137162	0.2583021
10.000	1.015625	0.05649523
15.000	1.032385	0.09267782
17.500	0.9850001	0.04876236
20.000	1.27377	0.07567287
22.500	0.9457317	0.06276392
25.000	1.081034	0.09664015
27.500	1.169388	0.06053901
30.000	0.9308418	0.05079515

Table S7 b.

Mean	1.063
STD	0.1129
SEM	0.03765

Table S8. Calculation of a correction factor for SLFA following AUI sampling mimicking $LESA_{ligand}$ sampling. The absorbance of samples following AUI sampling resembling $LESA_{ligand}$ sampling were compared with the absorbance of reference solutions at each different concentration (triplicate measurements); mean values ± SEM are given (**a**). The mean correction factor value from nine different concentrations is shown with the corresponding one STD and SEM (**b**) that were used as the correction factor ± STD for L_0 of SLFA in an attempt for calculating the K_d

Table S8 a.

	Corr. = $LESA_{ligand}$ / ref. at $Abs._{258\ nm}$	
Concentration (µM)	Mean	SEM
2.500	1.031746	0.2767644
3.500	0.8755102	0.1966019
5.000	0.5884956	0.1044601
7.500	0.8452139	0.1354036
10.000	0.9402986	0.130884
15.000	0.9828025	0.1384247
20.000	1.137392	0.1003392
25.000	1.219858	0.07775928
30.000	1.291995	0.1190583

Table S8 b.

Mean	0.9904
STD	0.2137
SEM	0.07123

Table S9. Calculation of a correction factor for CTZ following AUI sampling closely mimicking $LESA_{ligand}$. The absorbance of samples following AUI sampling resembling $LESA_{ligand}$ sampling were compared with the absorbance of reference solutions at each different concentration (triplicate measurements); mean values and SEM are given (**a**). The mean correction value from eight different concentrations is shown with the corresponding ± SEM (**b**) that were used as the correction factor ± STD for L_0 of CTZ in an attempt of calculating K_d respectively.

Table S9 a.

	Corr. = $LESA_{ligand}$ / ref. at $Abs._{277\ nm}$	
Concentration (µM)	Mean	SEM
10.000	1.860287	0.4149191
12.500	1.492	0.3896415
15.000	1.416981	0.1589581
17.500	1.544753	0.2254834
20.000	1.448179	0.2130674
22.500	1.314252	0.1998888

25.000	1.612676	0.2316224
30.000	1.60495	0.1470741

Table S9 b.

Mean	1.537
STD	0.1643
SEM	0.05807

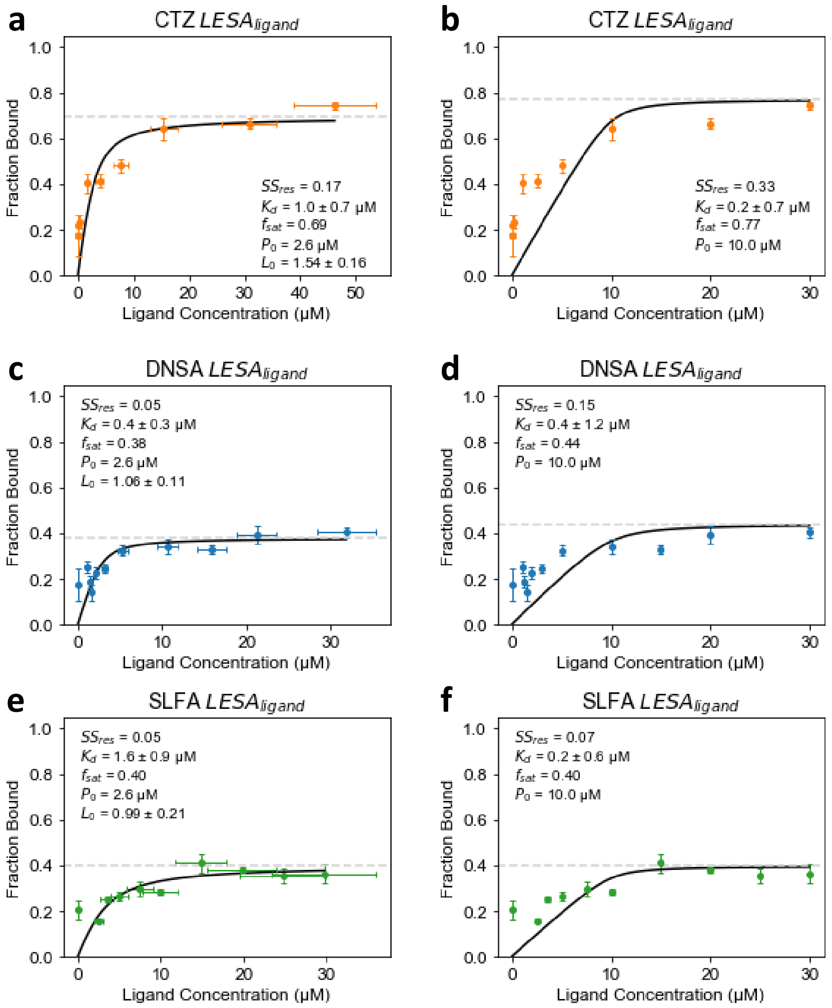

Fig S14. Titration plots of CTZ including a correction for both P_0 and L_0 as shown in the bottom right corner (**a**), and without corrections to determine corresponding K_ds from the fits (**b**) following $LESA_{ligand}$. Titration plots of DNSA including corrections both for P_0 and L_0 (**c**), and without any corrections for P_0 and L_0 during K_d calculations (**d**) following $LESA_{ligand}$. Titration plots of SLFA including corrections for both P_0 and L_0 (**e**), and SLFA with no corrections made for P_0 and L_0 (**f**) after $LESA_{ligand}$. All experimental data points represent mean values ± STD, experimental data included all three charge states of CAH in the absence of presence of a given ligand, following $LESA_{ligand}$ sampling, n=3; the black lines show the fit aiding determination of K_ds. SS_{res} indicates the goodness of fit. f_{sat} is shown for each plot. The STD of L_0 was propagated across the x axis where corrections were made for L_0 in an attempt to examine the resultant K_ds.

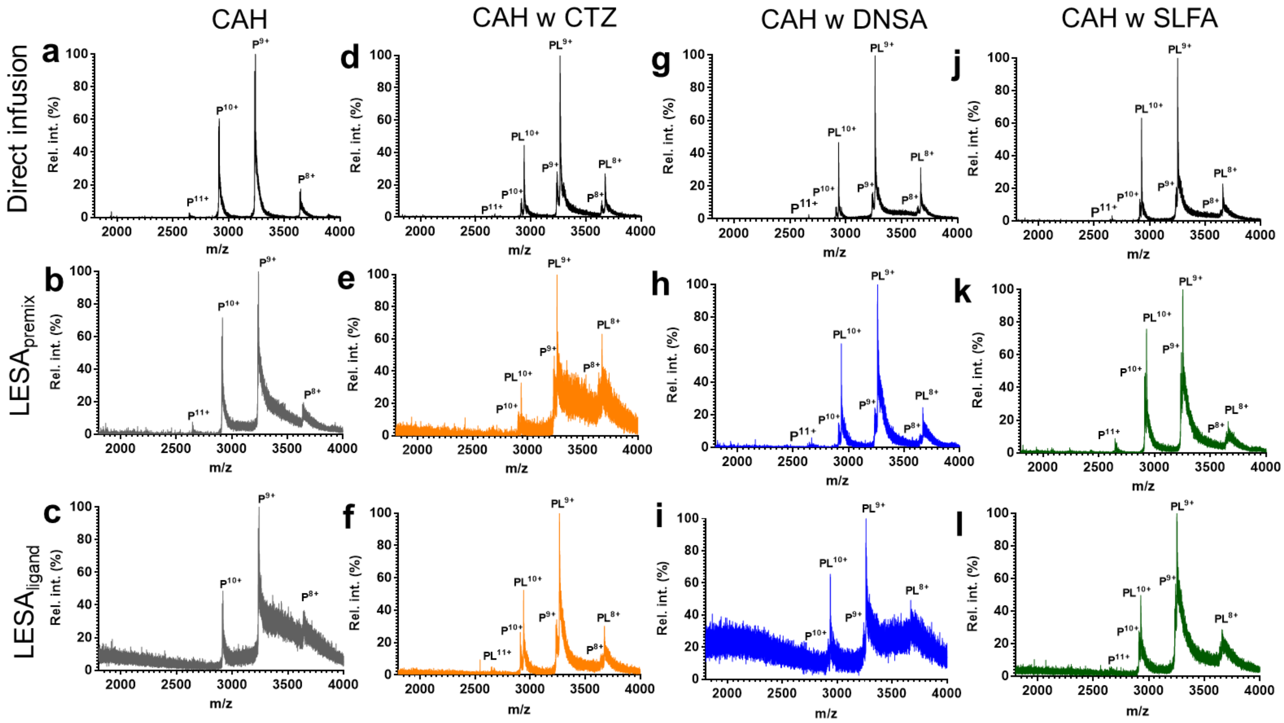

Fig S15. Mass spectra of CAH obtained in direct infusion mode (**a**), following $LESA_{premix}$ (**b**) and $LESA_{ligand}$ (**c**). Mass spectra of CAH in the presence of 30 µM CTZ acquired in direct infusion mode (**d**), following $LESA_{premix}$ sampling (**e**) and $LESA_{ligand}$ (**f**). Mass spectra of CAH in the presence of DNSA acquired by infusion (**g**), CAH with DNSA obtained using $LESA_{premix}$ sampling (**h**) and CAH in the presence of DNSA following $LESA_{ligand}$ (**i**). Mass spectra of CAH in the presence of 30 µM SLFA acquired in direct infusion (**j**), following $LESA_{premix}$ sampling (**k**), and $LESA_{ligand}$ sampling (**l**). All mass spectra were obtained in 25 mM ammonium acetate at pH 7.0 at 36 V wave height on a Synapt G2 S instrument in sensitivity mode; the ligand concentrations indicate uncorrected ligand concentrations.

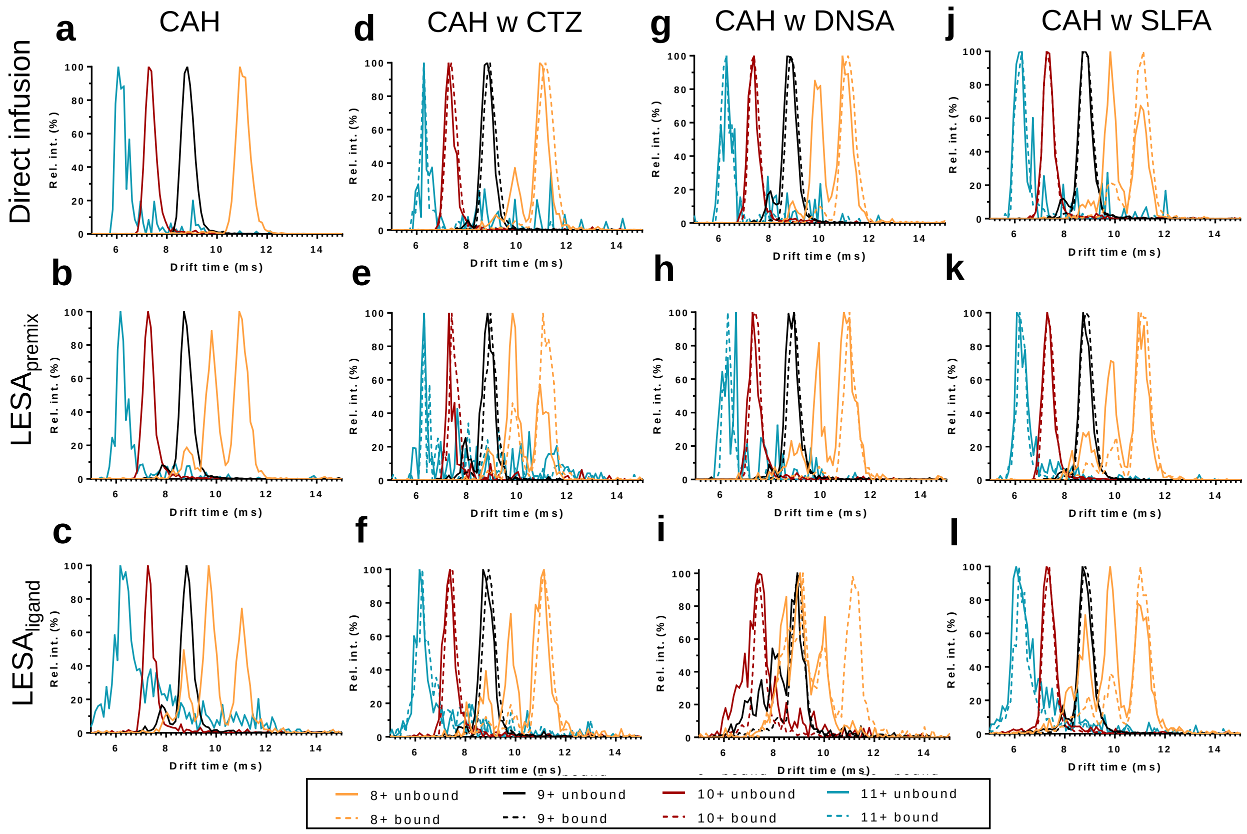

Fig S16. Drift time distribution profiles of CAH only in infusion mode (**a**), CAH only in LESA$_{premix}$ mode (**b**), and LESA$_{ligand}$ mode (**c**). Drift time profile distribution of CAH in the presence of CTZ acquired in infusion mode (**d**) including both ligand bound and unbound ions, drift time profile of CAH in the presence of CTZ following LESA$_{premix}$ sampling (**e**), and LESA$_{ligand}$ sampling (**f**). Drift time profile of CAH with DNSA acquired following direct infusion (**g**), LESA$_{premix}$ (**h**), and LESA$_{ligand}$ sampling (**i**). Drift time distribution profile of CAH in the presence of SLFA in infusion mode (**j**), following LESA$_{premix}$ (**k**) and LESA$_{ligand}$ sampling (**m**). Orange continuous line: 8^+ unbound ions, orange discontinuous line 8^+ bound ions, black continuous line 9^+ unbound ions, black discontinuous line 9^+ bound ions, purple continuous line 10^+ unbound ions, purple discontinuous ions 10^+ bound ions, blue continuous line 11^+ unbound ions and blue discontinuous line 11^+ bound ions; 36 V wave height.

Table S10. Mean $^{TW}CCS_{N2\rightarrow He}$ ± STD of CAH in the presence or absence of ligands under native-like conditions in 25 mM ammonium acetate at pH 7.0 with 1 STD, n=3. Data were acquired on a Synapt G2 S mass spectrometer in positive ionisation, in sensitivity mode. The literature CCSs for CAH only exhibit a wide spread depending on the gas in which the CCS was determined, the type of sample preparation and instrumentation as discussed before in more detail previously[12].

**Protein +/- ligand**	**z**	**$^{TW}CCS_{N2\rightarrow He}$ ± STD inf. (Å^2)**		**$^{TW}CCS_{N2\rightarrow He}$ ± STD LESA premix (Å^2)**		**$^{TW}CCS_{N2\rightarrow He}$ ± STD LESA ligand (Å^2)**		**Literature $^{TW}CCS_{N2\rightarrow He}$ (Å^2)**
		unbound-	bound-	unbound-	bound-	unbound-	bound-	
10 µM CAH	8+	**2366 ± 46**		**2361 ± 52**		**2376 ± 51**		
		-		**2266 ± 37**		**2196 ± 40**		
		-		**2206 ± 46**		**2065 ± 33**		
	9+	**2331 ± 38**		**2313 ± 37**		**2325 ± 46**		**2004 ± 8, $N_2\rightarrow He$[13]<br>2425 ± 12, $N_2\rightarrow He$[14]<br>2309 ± 23 and 2520 ± 28, N_2[14]**
		-		**2172 ± 23**		**2166 ± 26**		
	10+	**2295 ± 33**		**2295 ± 33**		**2295 ± 33**		**2003 ± 26, $N_2\rightarrow He$[13]<br>2203 ± 26, $N_2\rightarrow He$[13]<br>2491 ± 20, $N_2\rightarrow He$[14]<br>2580 ± 36, N_2[14]<br>2430, He[15]**
	11+	**2278 ± 15**		**2296 ± 28**		**2279 ± 34**		**2048 ± 24, $N_2\rightarrow He$[13]<br>2558 ± 23, $N_2\rightarrow He$[14]<br>2615 ± 31, N_2[14]<br>2430, He[15]**
10 µM CAH 30 µM CTZ	8+	**2361 ± 52**	**2391 ± 52**	**2366 ± 46**	**2380 ± 51**	**2371 ± 59**	**2391 ± 54**	
		**2217 ± 50**	**2222 ± 46**	**2216 ± 38**	**2226 ± 38**	**2206 ± 47**	**2216 ± 38**	
		**2093 ± 57**	-	**2076 ± 28**	**2087 ± 50**	**2060 ± 16**	**2081 ± 25**	
	9+	**2325 ± 41**	**2342 ± 47**	**2325 ± 41**	**2348 ± 39**	**2319 ± 30**	**2348 ± 39**	
		**2185 ± 45**	-	**2184 ± 33**	**2196 ± 24**	**2165 ± 32**	**2196 ± 24**	
	10+	**2301 ± 34**	**2316 ± 38**	**2295 ± 33**	**2323 ± 24**	**2309 ± 44**	**2323 ± 35**	
	11+	**2296 ± 32**	**2314 ± 18**	**2297 ± 29**	**2305 ± 20**	**2305 ± 35**	**2321 ± 24**	
10 µM CAH 30 µM DNSA	8+	**2381 ± 62**	**2386 ± 57**	**2384 ± 35**	**2391 ± 54**	**2404 ± 41**	**2395 ± 49**	
		**2222 ± 60**	**2237 ± 48**	**2230 ± 45**	**2231 ± 39**	**2242 ± 43**	**2227 ± 43**	
		**2072 ± 46**	-	**2075 ± 32**	**2121 ± 38**	**2098 ± 39**	**2103 ± 51**	
	9+	**2331 ± 56**	**2337 ± 49**	**2331 ± 56**	**2342 ± 47**	**2337 ± 48**	**2348 ± 39**	
		**2197 ± 35**	**2197 ± 35**	**2184 ± 33**	**2197 ± 53**	**2184 ± 33**	**2215 ± 43**	
	10+	**2309 ± 37**	**2316 ± 34**	**2295 ± 33**	**2316 ± 33**	-	-	
	11+	**2304 ± 34**	**2304 ± 34**	**2280 ± 45**	**2313 ± 20**	-	-	
10 µM CAH 30 µM SLFA	8+	**2375 ± 58**	**2391 ± 54**	**2365 ± 50**	**2371 ± 55**	**2315 ± 115**	**2376 ± 53**	
		**2206 ± 47**	**2216 ± 38**	**2221 ± 32**	**2226 ± 61**	**2168 ± 91**	**2206 ± 53**	
		**2077 ± 43**	**2108 ± 45**	**2081 ± 50**	**2086 ± 25**	**2057 ± 62**	**2096 ± 26**	
	9+	**2319 ± 48**	**2324 ± 27**	**2313 ± 37**	**2325 ± 41**	**2330 ± 20**	**2336 ± 31**	
		**2175 ± 36**	**2137 ± 72**	**2185 ±45**	**2191 ± 43**	**2185 ± 28**	**2209 24**	
	10+	**2302± 24**	**2302± 24**	**2295 ± 33**	**2302 ± 24**	**2301 ± 34**	**2316 ± 34**	
	11+	**2297 ± 29**	**2296 ± 32**	**2270 ± 5**	**2279 ± 20**	-	-	

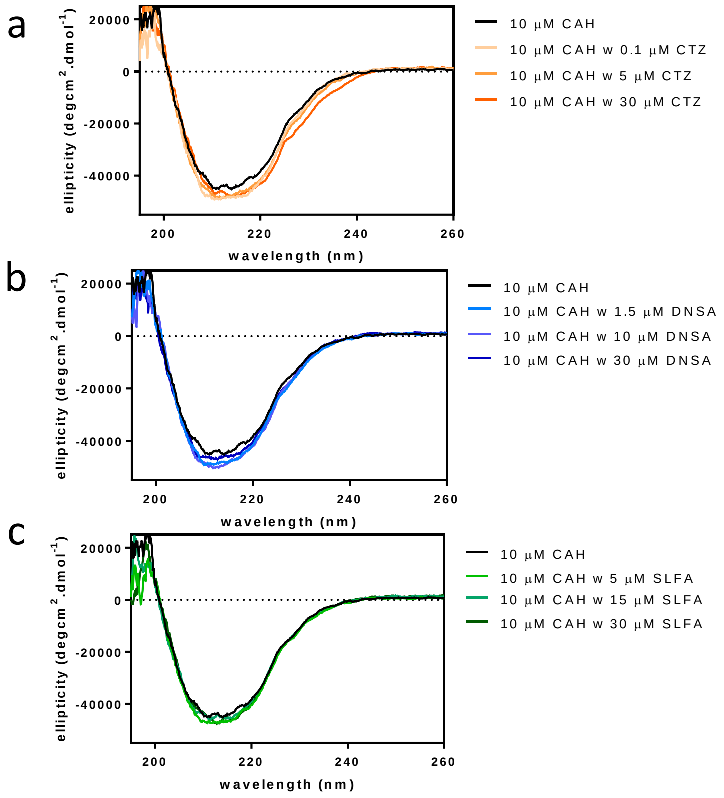

Fig S17. CD spectra of CAH in the absence or presence of CTZ (0.1 µM, 5 µM and 30 µM) (a), CD spectra of CAH in the absence or presence of DNSA (1.5 µM, 10 µM and 30 µM) (b) and CD spectra of CAH in the absence or presence of SLFA (1.5 µM, 15 µM and 30 µM).

References

1. Myszka, D. G.; Abdiche, Y. N.; Arisaka, F.; Byron, O.; Eisenstein, E.; Hensley, P.; Thomson, J. A.; Lombardo, C. R.; Schwarz, F.; Stafford, W.; Doyle, M. L., The ABRF-MIRG'02 study: assembly state, thermodynamic, and kinetic analysis of an enzyme/inhibitor interaction. *Journal of biomolecular techniques : JBT* **2003,** *14* (4), 247-269.
2. Bootorabi, F.; Jänis, J.; Valjakka, J.; Isoniemi, S.; Vainiotalo, P.; Vullo, D.; Supuran, C. T.; Waheed, A.; Sly, W. S.; Niemelä, O.; Parkkila, S., Modification of carbonic anhydrase II with acetaldehyde, the first metabolite of ethanol, leads to decreased enzyme activity. *BMC Biochem.* **2008,** *9* (1), 32.
3. Ruotolo, B. T.; Benesch, J. L.; Sandercock, A. M.; Hyung, S. J.; Robinson, C. V., Ion mobility-mass spectrometry analysis of large protein complexes. *Nat. Protoc.* **2008,** *3* (7), 1139-1152.
4. Bush, M. F.; Hall, Z.; Giles, K.; Hoyes, J.; Robinson, C. V.; Ruotolo, B. T., Collision Cross Sections of Proteins and Their Complexes: A Calibration Framework and Database for Gas-Phase Structural Biology. *Anal. Chem.* **2010,** *82* (22), 9557-9565.
5. Shelimov, K. B.; Clemmer, D. E.; Hudgins, R. R.; Jarrold, M. F., Protein Structure in Vacuo: Gas-Phase Conformations of BPTI and Cytochrome c. *J. Am. Chem. Soc.* **1997,** *119* (9), 2240-2248.
6. Zhang, Z.; Marshall, A. G., A universal algorithm for fast and automated charge state deconvolution of electrospray mass-to-charge ratio spectra. *J. Am. Soc. Mass. Spectrom.* **1998,** *9* (3), 225-233.
7. Gudiksen, K. L.; Urbach, A. R.; Gitlin, I.; Yang, J.; Vazquez, J. A.; Costello, C. E.; Whitesides, G. M., Influence of the Zn(II) Cofactor on the Refolding of Bovine Carbonic Anhydrase after Denaturation with Sodium Dodecyl Sulfate. *Anal. Chem.* **2004,** *76* (24), 7151-7161.
8. Schachner, L. F.; Ives, A. N.; McGee, J. P.; Melani, R. D.; Kafader, J. O.; Compton, P. D.; Patrie, S. M.; Kelleher, N. L., Standard Proteoforms and Their Complexes for Native Mass Spectrometry. *J. Am. Soc. Mass. Spectrom.* **2019,** *30* (7), 1190-1198.
9. Day, Y. S.; Baird, C. L.; Rich, R. L.; Myszka, D. G., Direct comparison of binding equilibrium, thermodynamic, and rate constants determined by surface- and solution-based biophysical methods. *Protein Sci.* **2002,** *11* (5), 1017-1025.
10. Jecklin, M. C.; Schauer, S.; Dumelin, C. E.; Zenobi, R., Label-free determination of protein-ligand binding constants using mass spectrometry and validation using surface plasmon resonance and isothermal titration calorimetry. *Journal of molecular recognition : JMR* **2009,** *22* (4), 319-329.
11. Cannon, M. J.; Papalia, G. A.; Navratilova, I.; Fisher, R. J.; Roberts, L. R.; Worthy, K. M.; Stephen, A. G.; Marchesini, G. R.; Collins, E. J.; Casper, D.; Qiu, H.; Satpaev, D.; Liparoto, S. F.; Rice, D. A.; Gorshkova, I. I.; Darling, R. J.; Bennett, D. B.; Sekar, M.; Hommema, E.; Liang, A. M.; Day, E. S.; Inman, J.; Karlicek, S. M.; Ullrich, S. J.; Hodges, D.; Chu, T.; Sullivan, E.; Simpson, J.; Rafique, A.; Luginbühl, B.; Westin, S. N.; Bynum, M.; Cachia, P.; Li, Y.-J.; Kao, D.; Neurauter, A.; Wong, M.; Swanson, M.; Myszka, D. G., Comparative analyses of a small molecule/enzyme interaction by multiple users of Biacore technology. *Anal. Biochem.* **2004,** *330* (1), 98-113.
12. Illes-Toth, E.; Cooper, H. J., Probing the Fundamentals of Native Liquid Extraction Surface Analysis Mass Spectrometry of Proteins: Can Proteins Refold during Extraction? *Anal. Chem.* **2019,** *91* (19), 12246-12254.
13. Leary, J. A.; Schenauer, M. R.; Stefanescu, R.; Andaya, A.; Ruotolo, B. T.; Robinson, C. V.; Thalassinos, K.; Scrivens, J. H.; Sokabe, M.; Hershey, J. W. B., Methodology for measuring conformation of solvent-disrupted protein subunits using T-WAVE ion mobility MS: An investigation into eukaryotic initiation factors. *J. Am. Soc. Mass Spectrom.* **2009,** *20* (9), 1699-1706.
14. Harrison, J. A.; Kelso, C.; Pukala, T. L.; Beck, J. L., Conditions for Analysis of Native Protein Structures Using Uniform Field Drift Tube Ion Mobility Mass Spectrometry and Characterization of Stable Calibrants for TWIM-MS. *J. Am. Soc. Mass. Spectrom.* **2019,** *30* (2), 256-267.

15. Allen, S. J.; Giles, K.; Gilbert, T.; Bush, M. F., Ion mobility mass spectrometry of peptide, protein, and protein complex ions using a radio-frequency confining drift cell. *Analyst* **2016,** *141* (3), 884-891.
