# Supplementary material for: Quantitative Characterization of Three Carbonic Anhydrase Inhibitors by LESA Mass Spectrometry
Source: J Am Soc Mass Spectrom. 2022 Jun 8;33(7):1168–75. doi: 10.1021/jasms.2c00024 (PMC9264382; doi:10.1021/jasms.2c00024)
Supplement: Supplementary file 2 — js2c00024_si_002.zip [file js2c00024_si_002.zip › Supplemental file1_FINAL_RESUB_FINAL/Supplemental file1_FINAL_revised_EKS/Supplementary Information File 1.docx]

Supplementary Information

Matlab Files

- **ForLoopedAUCAllChargeStates.m** – script for calculating the area under the protein and protein-ligand peaks using the trapezium rule. This script will require the user to have imzMLconverter available from <https://www.cs.bham.ac.uk/~ibs/imzMLConverter/> and their data to be summed and converted in .mzML format.

How to run

1. Download all the files
2. Change the following inputs in **ForLoopedAUCAllChargeStates.m**
   1. Path – location of .mzML spectra files
   2. imzMLConverterLocation – location of imzMLconverter
   3. m/z values of protein and protein ligand peaks

Python Files

- **CAH_paper_Kd_fitting.ipynb** – jupyter notebook for fitting KD curves

How to run

1. Download Anaconda (if not already installed on your system)
2. Open Anaconda navigator and launch Jupyter notebook and navigate to the location of the .ipynb file
3. Open notebook
4. Change the file paths (‘fnames’) to the location of the csv files created by the matlab code
